# Seeing the Invisibles: Detection of Peptide Enantiomers, Diastereomers, and Isobaric Ring Formation in Lanthipeptides Using Nanopores

**DOI:** 10.1021/jacs.3c04076

**Published:** 2023-08-14

**Authors:** Roderick Corstiaan Abraham Versloot, Patricia Arias-Orozco, Matthijs Jonathan Tadema, Florian Leonardus Rudolfus Lucas, Xinghong Zhao, Siewert J. Marrink, Oscar Paul Kuipers, Giovanni Maglia

**Affiliations:** Groningen Biomolecular Sciences and Biotechnology Institute, University of Groningen, 9747AG Groningen, Netherlands

## Abstract

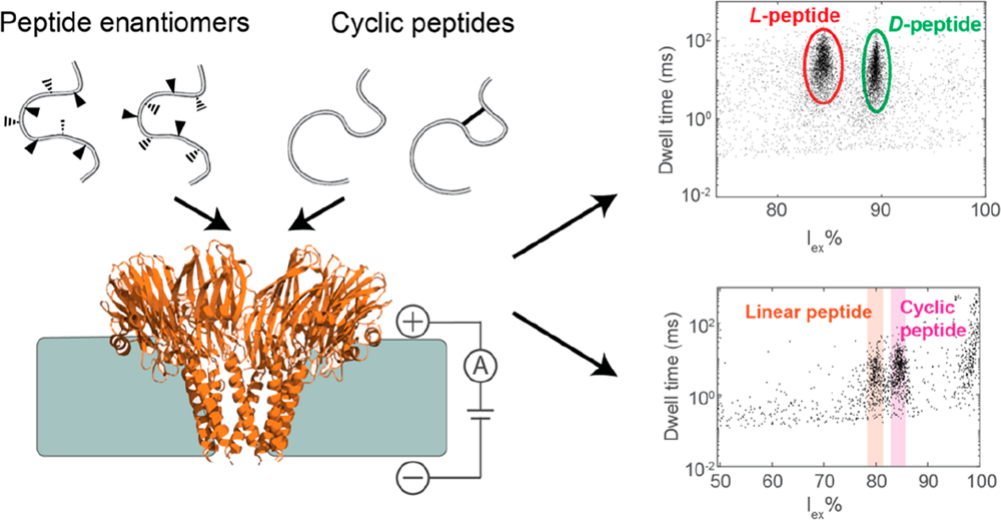

Mass spectrometry (MS) is widely used in proteomic analysis but cannot differentiate between molecules with the same mass-to-charge ratio. Nanopore technology might provide an alternative method for the rapid and cost-effective analysis and sequencing of proteins. In this study, we demonstrate that nanopore currents can distinguish between diastereomeric and enantiomeric differences in l- and d-peptides, not observed by conventional MS analysis, down to individual d-amino acids in small opioid peptides. Molecular dynamics simulations suggest that similar to chiral chromatography the resolution likely arises from multiple chiral interactions during peptide transport across the nanopore. Additionally, we used nanopore recordings to rapidly assess 4- and 11-amino acid ring formation in lanthipeptides, a process used in the synthesis of pharmaceutical peptides. The cyclization step requires distinguishing between constitutional isomers, which have identical MS signals and typically involve numerous tedious experiments to confirm. Hence, nanopore technology offers new possibilities for the rapid and cost-effective analysis of peptides, including those that cannot be easily differentiated by mass spectrometry.

## Introduction

Mass spectrometry (MS) is widely applied in the field of proteomics for its high accuracy and ability to measure complex mixtures.^1^ In MS, molecules are identified by their mass-to-charge ratio, a feature that can be used to resolve the molecular weight with sub-Dalton accuracy. This allows MS to separate peptides with different mass and/or charge but makes it challenging to directly discriminate between peptide features such as certain glycosylation isomers, the presence of d-amino acids, and ring formation in peptides.^2^ Most of these challenges can be addressed using additional chromatography techniques such as ion mobility mass spectrometry and ion exchange chromatography, where the ions are separated before the MS measurement.^3−7^ While this allows for differentiation between isomeric peptide features, these methods rely on small differences in the mobility of isomers that might not allow full separation. In addition, although tandem MS is routinely used to assess ring formation in short peptides, the fragmentation patterns from cyclized peptides can be tedious to analyze.^8^ Since both cyclic peptides and d-amino acid containing peptides are promising for the development of therapeutics, a direct analysis to replace or complement MS-based analyses of isomers would be desired.

Nanopore analysis is a promising single-molecule technique for the identification of a variety of molecules. One of the main advantages of nanopore technology is that ionic currents are easily integrated with silicon-based electronics, which will allow the manufacturing of portable and low-cost devices as substitutes for, or alongside, mass spectrometry analysis. The nanopore signal ultimately depends on the volume and interactions of the analyte with the nanopore rather than the mass of the analyte,^9−12^ providing a different physical principle for molecular discrimination compared to mass spectrometry.

Peptides have been detected by nanopores,^12−20^ including their post-translational modifications.^21−23^ It has also been shown that when the ionic and buffer conditions are accordingly selected, peptides with different charges and chemical composition can be identified, and the nanopore signal is related to the size (volume) of the peptide analyzed.^24^ Using optimized conditions and engineered nanopores, a method reminiscent of early MS methods was developed where proteins are identified by the peptide spectral analysis.^19,20^ Nanopores have also been used to discriminate between scrambled model peptides and peptides with isomeric modifications that were trapped in aerolysin nanopores,^25,26^ although such peptides could already be differentiated by tandem mass spectrometry. Recently, OmpF nanopores, holding a monomeric sensing region, were shown to detect differences in two enantiomers in highly charged β-amyloid peptides.^27^ It was suggested that the exact positioning of electrostatic interactions in the asymmetric monomeric nanopore was important for chiral recognition.

Here, we demonstrate the ability of two different biological nanopores to detect differences in several types of isomeric peptides, including constitutional isomers, enantiomers, and diastereomers that cannot be directly discriminated by conventional mass spectrometry. We show that nanopores with different structures (an α-helical or β-strand recognition region), oligomeric-fold symmetry, and chemical composition can distinguish the peptides. MD simulations revealed that rather than specific interactions between the nanopore and the peptide, the difference in the signal is caused by multiple chiral interactions within the lumen of the nanopore when traversing the nanopore. Hence, similar to chiral chromatography, in principle, all nanopores should be capable of resolving chiral differences in molecules. Then, we investigate lanthipeptide ring formation, a real-word problem in the synthesis of pharmaceutical peptides.^28,29^ Since this process cannot be directly followed by conventional mass spectrometry, it typically requires a lengthy procedure to be established. Nanopore analysis provided quantitative analysis of ring formation within minutes. This work shows that nanopore experiments are promising tools for identifying and characterizing isobaric peptide modifications in the development of therapeutic and antimicrobial peptides.

## Results and Discussion

### Discrimination between Homochiral Peptides in CytK and FraC Nanopores

**Figure 1:**
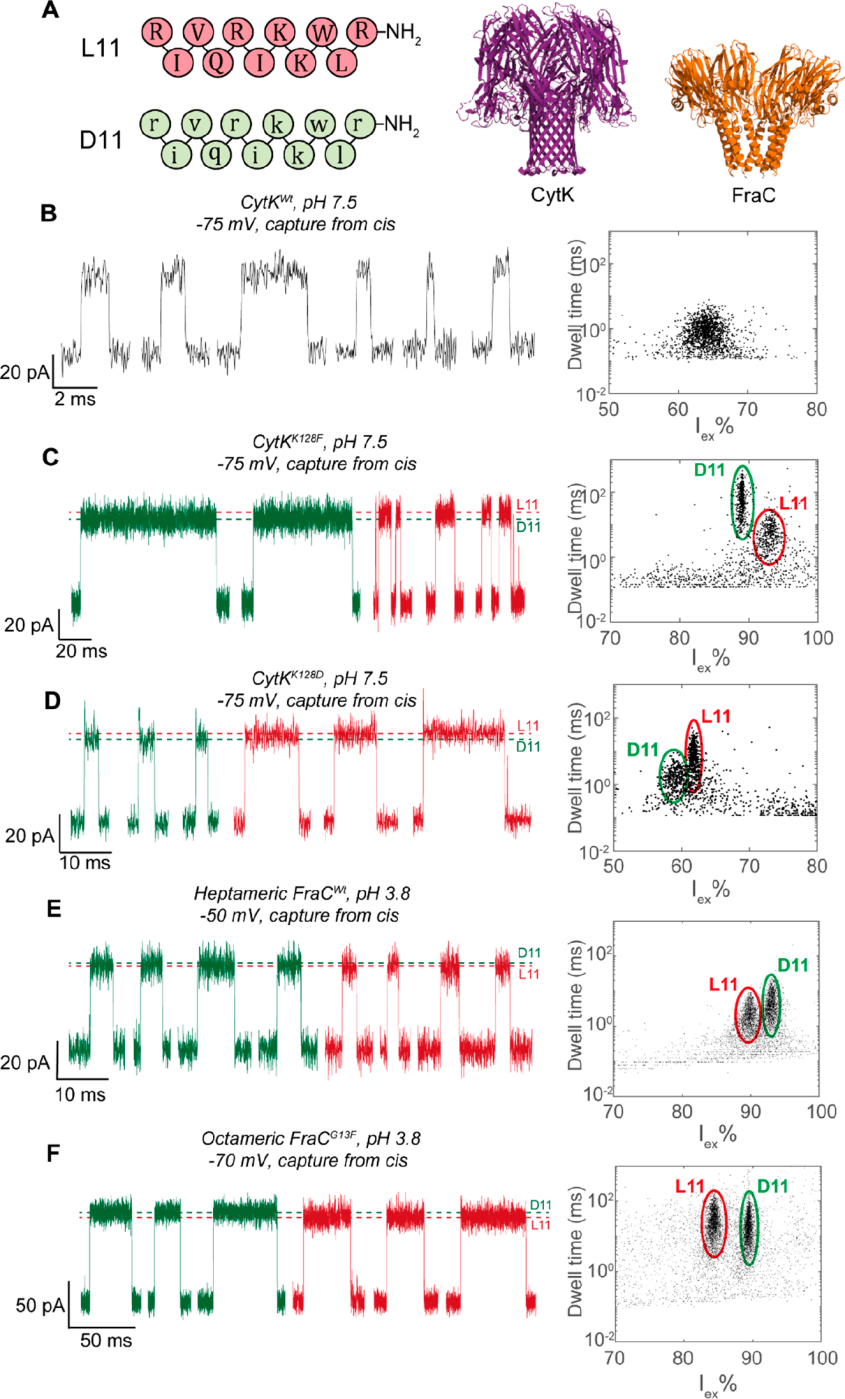
Detection of D11 and L11 in CytK and FraC nanopores. (A) Amino acid composition of L11 and D11 (left) and cartoon representation of the crystal structures of cytotoxin K (CytK) and fragaceatoxin C (FraC, PDB: 4TSY) nanopores (right). The crystal structure of CytK is based on homology modeling to α-hemolysin (PDB: 7AHL). (B–F) Typical ionic current blockades (left) and scatter plots showing event characteristics (right) of an equimolar mixture of 2.5 μM D11 and 2.5 μM L11 in (B) CytK^Wt^, (C) CytK^K128F^, (D) CytK^K128D^, (E) FraC^Wt^, and (F) FraC^G13F^. Events belonging to L11 and D11 are marked in red and green, respectively. For the recordings, measurements were taken in 1 M KCl at pH 7.5 (CytK) or 3.8 (FraC) with a sampling rate of 50 kHz and a 10 kHz Bessel filter.

To test the ability of nanopores to identify differences in isobaric peptides invisible to MS analysis, we first tested 2 enantiomeric 11-residue peptides consisting entirely of either d- or l*-*amino acids (D11 and L11, respectively, Figure 1A) that were previously used to enhance antibiotic activity against Gram-negative bacteria.^30^ We tested β-barrel CytK and α-helical FraC nanopores, which showed peptide detection in earlier work.^31,32^ The nanopore and the ionic current blockades were characterized by their dwell time and excluded current (*I*_ex_% = (*I*_O_ – *I*_B_)/*I*_O_ × 100%, where *I*_O_ is the open pore current and *I*_B_ is the average ionic current during the event). The addition of a 1:1 mixture of both peptides to CytK nanopores revealed that the wild-type pore is unable to discriminate between the d- and l-peptide enantiomers at pH 7.5 (Figure 1B) or pH 3.8 (Figure S1 and Figure S2). Voltage dependency measurements of individual peptides showed that no significant differences in *I*_ex_% and dwell times were observed under a range of voltages between −60 and −140 mV (Figure S3). However, the difference between D11 and L11 could be detected in CytK^K128F^ (Figure 1C) and CytK^K128D^ (Figure 1D) nanopores, two mutants with an aromatic or acidic sensing region, respectively, isolated for enhanced peptide analysis, where the d-peptide induced smaller blockades than did the l-peptide (Figures S3 and S4). The two enantiomeric peptides could also be observed using α-helical FraC^Wt^ (Figure 1E) and FraC^G13F^ nanopores (Figure 1F). However, the d-peptide had an excluded current higher than that of the l-peptide.

### Detection Mechanism of Chirality in CytK Nanopores

**Figure 2:**
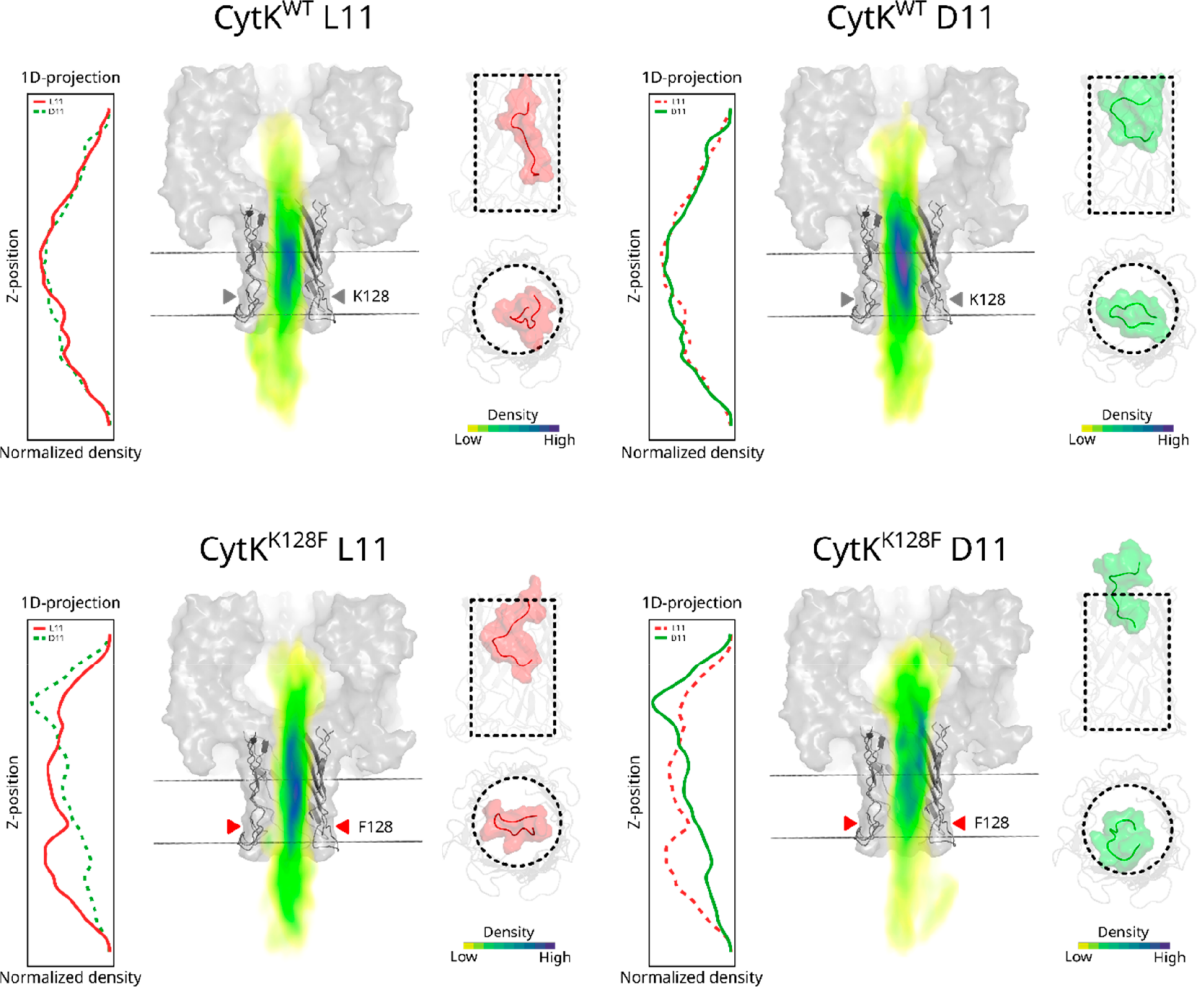
Molecular dynamics simulation of L11 and D11 in CytK nanopores. Each combination of pore and peptide shows a normalized volumetric map colored by the probability density associated with that position. Also shown are representative structures of the simulated pore barrel and an overlay of the entire pore in a schematic membrane for reference. The graph to the left shows the probability density along the pore axis, and the graph on the right is the highest probability frame of each simulation to illustrate the positioning of the peptide in the barrel. The gray and red triangles indicate the position of residue 128 along the β barrel.

To explore the molecular mechanism behind the discrimination between D11 and L11 peptides, we used all-atom molecular dynamics simulations to probe the peptide translocation events. To keep the simulations feasible, the model of the nanopore included only the barrel-forming residues, and the shape of the barrel was kept in place by gentle positional restraints to the reference structure; the fluctuations in the backbone geometry were captured by tuning the restraints to match the flexibility of the full barrel in the membrane. The peptide structure was generated in a linear conformation and aligned with the pore with the N-terminus pointing down to mimic the alignment with the electric field that would be present in electrophysiology experiments (Figure S5). Well-tempered metadynamics, used to speed up the sampling of the relevant degrees of freedom,^33^ was used to simulate peptide translocation events and the interactions of both D11 and L11 in CytK^Wt^ and CytK^K128F^ nanopores. The resulting energy landscapes (Figure S6) contain distinct energy minima and barriers that are different between the pore variants and the two isomeric peptides. The general shape of the landscape showed an energy minimum within the pore lumen, a barrier at the pore entrance, and a barrier at the exit of the pore,^34^ as shown by the volumetric density map overlaid with the structure of the CytK nanopore (Figure 2).

In CytK^Wt^ pores, the energy density maps of D11 and L11 along the β-barrel are very similar, with the highest density in the middle part of the barrel, suggesting that both peptides likely reside at similar locations in the pore. This observation agrees with the similar ionic current characteristics of D11 and L11 events in the nanopore measurements. In contrast, D11 peptides in CytK^K128F^ show a higher density at the entrance of the β-barrel than L11 peptides, indicating that the L11 peptide preferentially resides deeper in the nanopore than the D11 peptide. This might explain the difference in the nanopore translocation events that are observed in CytK^K128F^ nanopores. Further analysis of the simulations reveals that in CytK^Wt^ both peptides have a similar global energy minimum in terms of position and radius of gyration, whereas in CytK^K128F^ the minimum of L11 seems to shift toward a lower radius of gyration and D11 shifts toward a higher radius of gyration, corresponding to a more compact and a more linear conformation, respectively (Figure S6). If L11 tends toward a more compact conformation in the pore than D11, then it would explain why the excluded current of L11 is higher than that of D11. The interactions that underlie this phenomenon must be chiral in nature, but they are difficult to pin down. Rather, as observed in chiral chromatography, it is likely the sum of many chiral interactions that changes slightly due to the mutation that causes the observed effect.

### Detection of d-Amino Acids in Heterochiral Peptides

**Figure 3:**
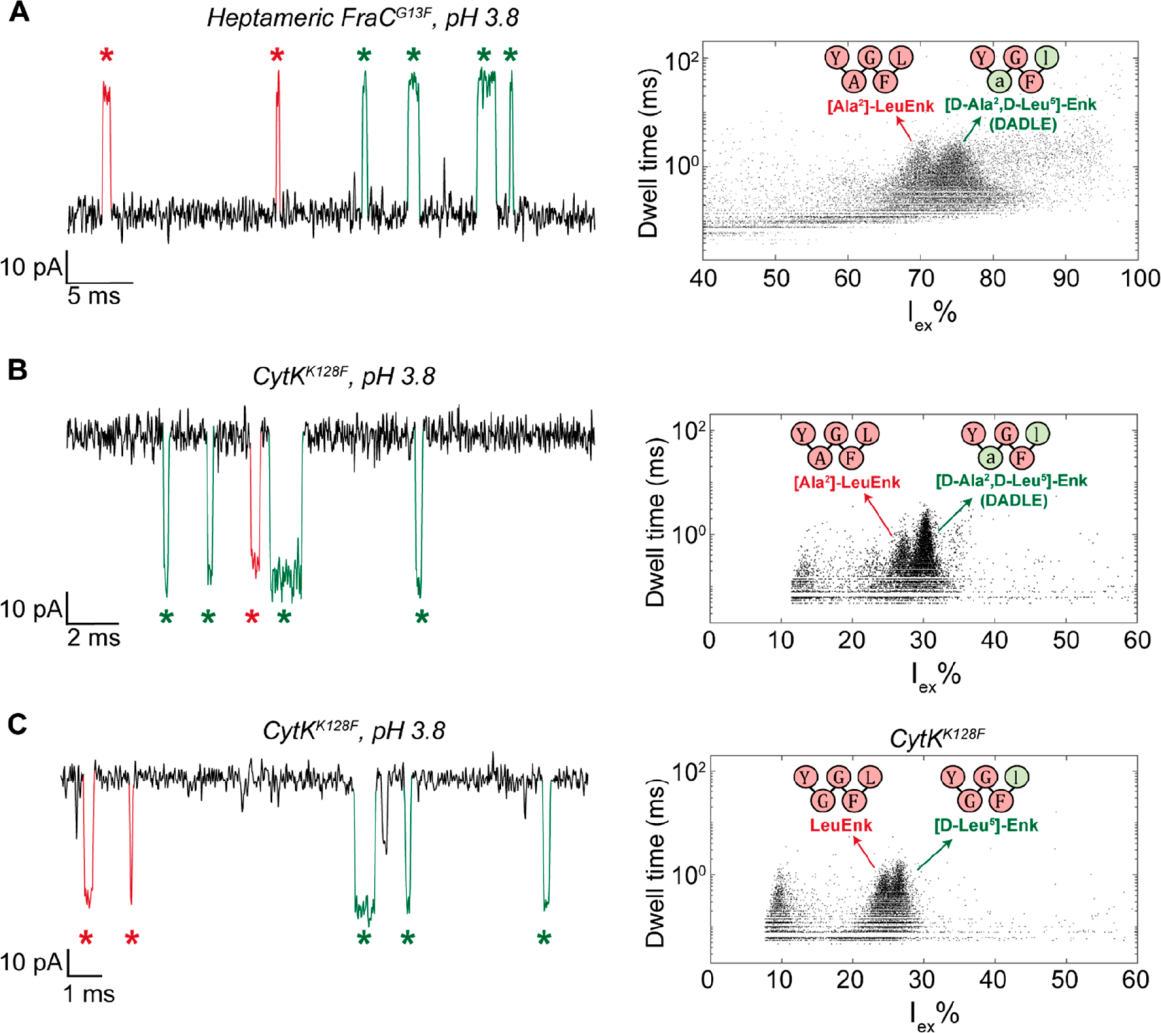
Detection of enkephalin peptides in FraC and CytK nanopores. (A) Ionic current trace and nanopore spectrum of a FraC^G13F^ nanopore after the addition of 10 μM [Ala^2^]-leucine enkephalin and 10 μM DADLE added to the cis chamber in 3 M LiCl at pH 3.8 with an applied voltage of −70 mV. (B) Ionic current trace and nanopore spectrum of a CytK^K128F^ nanopore after the addition of 2.5 μM [Ala^2^]-leucine enkephalin and 2.5 μM DADLE added to the trans chamber in 3 M LiCl at pH 3.8, +100 mV applied voltage. (C) Ionic current trace and nanopore spectrum of a CytK^K128F^ nanopore after the addition of 5 μM leucine enkephalin and 5 μM [d-Leu^5^]-enkephalin added to the trans chamber in 3 M LiCl at pH 3.8, +100 mV applied voltage. Events belonging to L11 and D11 are marked in red and green, respectively. Measurements were taken at a sampling rate of 50 kHz with a 10 kHz Bessel filter.

We further explored the ability of nanopores to distinguish between l- and d-peptides by testing small, neutral diastereomeric peptides. We first tested the delta opioid peptide DADLE ([d-Ala2,d-Leu5] enkephalin, or YdAGFdL), which contains a mixture of d- and l-amino acids. Thus, we compared the nanopore signal of DADLE to the l-amino acid counterpart ([Ala2] Leu-enkephalin or YAGFL). The peptides are small (569 Da) but can be accurately detected in heptameric FraC^G13F^ (Figure 3A) and CytK^K128F^ (Figure 3B) nanopores in high ionic strength (3 M LiCl) solutions.^23^ We measured a small difference in excluded currents in FraC^G13F^ (70.8 ± 2.6 *I*_ex_% for YAGFL and 74.4 ± 2.3 *I*_ex_% for YdAGFdL) and CytK^K128F^ (27.1 ± 1.1 *I*_ex_% for YAGFL and 30.2 ± 0.9 *I*_ex_% for YdGFdL). In order to quantify the resolution of the nanopore, we calculated the separation of the event clusters as
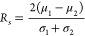
1where μ_1_ and μ_2_ are the centers (mean) and σ_1_ and σ_2_ are the spreads (standard deviations) of the peptide clusters as determined from Gaussian fitting to the *I*_ex_% histogram of individual peptide measurements (Figure S7).^31^ A higher **R_s_** indicates a better separation between the peptides, and a value of at least 2 is required to achieve more than 84% separation of the peptides. The *R_s_* values for FraC^G13F^ and CytK^K128F^ are 1.47 and 3.10, respectively (Table S1), indicating that in CytK^K128F^ in particular the clusters are well separated with only a 6% overlap. We used this pore to measure a second set of diastereomeric peptides, leucine enkephalin (YGGFL) and [d-Leu^5^]-enkephalin (YGGFdL), where only one amino acid is different. The two peptides could be differentiated by their difference in excluded current (24.5 ± 1.1 *I*_ex_% for leucine enkephalin versus 26.3 ± 0.9 *I*_ex_% for [d-Leu^5^]-enkephalin, Figure 3C), albeit with some overlap between the clusters (*R_s_* = 1.80 or 82% separation, Table S1). Nevertheless, these results show that our nanopore system is sensitive enough to detect a chiral difference of a single amino acid in peptides.

### Detection of Lanthipeptide Ring Formation in FraC Nanopores

**Figure 4:**
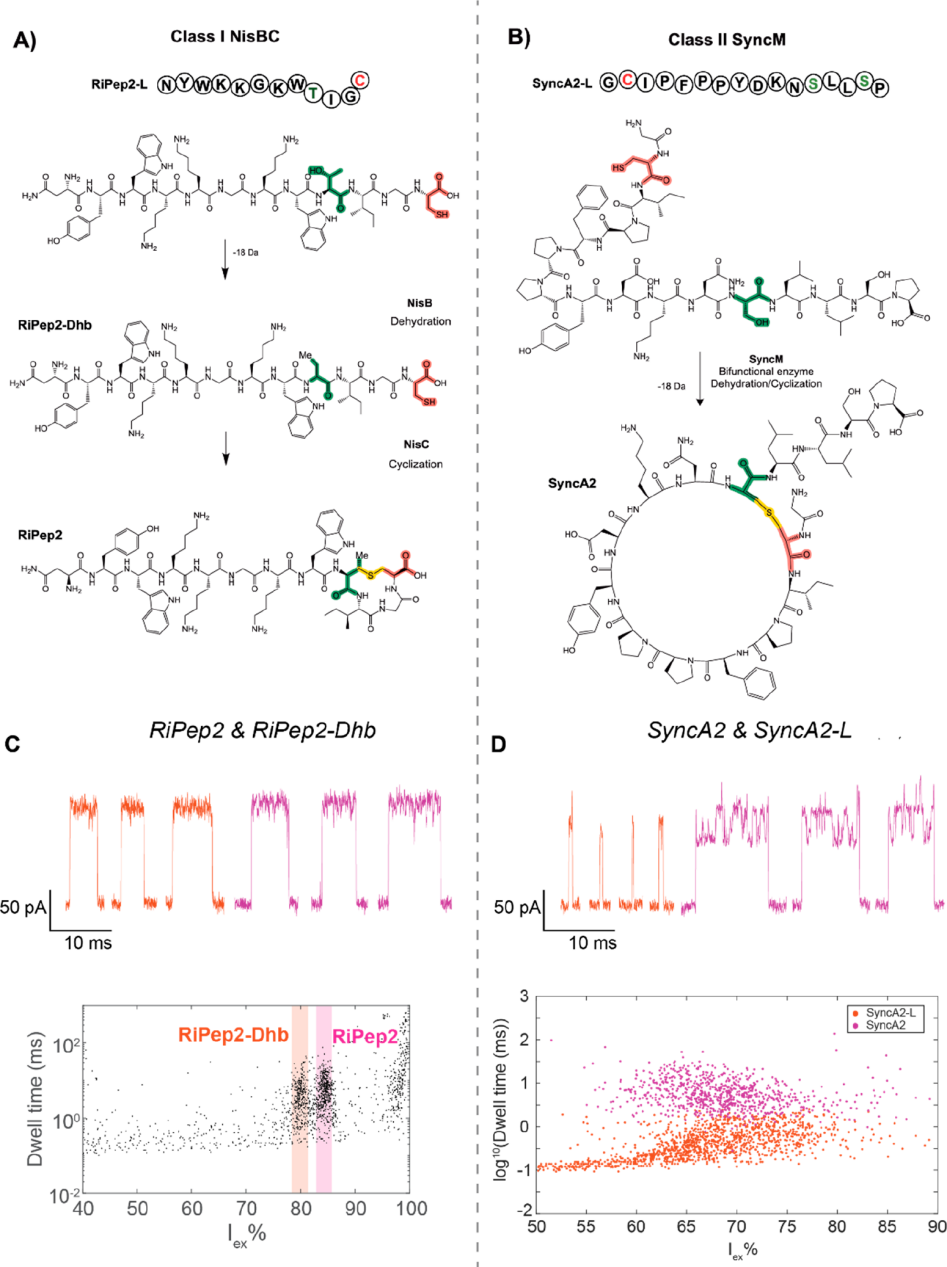
Detection of lanthipeptides in FraC nanopores. (A) Schematic representation of the cyclization of the RiPep2 peptide, following a dehydration step catalyzed by NisB and a cyclization step catalyzed by NisC. (B) Schematic representation of the one-step cyclization of SyncA2 from linear derivative SyncA2-L. (C) Typical nanopore events (top) and event spectrum (bottom) after the addition of a mixture of RiPep2-Dhb and RiPep2 to a FraC^G13F^ nanopore in 1 M KCl buffer at pH 3.8, −100 mV applied voltage. The *I*_ex_% ranges belonging to RiPep2-Dhb and RiPep are indicated in orange and purple, respectively. (D) Typical nanopore events (top) and event spectrum (bottom) after the addition of a mixture of SyncA2-L and SyncA2 to a FraC^Wt^ nanopore in 1 M KCl buffer at pH 3.8, −70 mV applied voltage. The events for SyncA2-L and SyncA2 as classified by logistics regression are shown in purple and orange, respectively.

We then used nanopores to detect ring formation in lanthipeptides, which requires distinguishing between two constitutional isomers, a process not detectable by MS analysis. We selected two lanthipeptides derived from *L. lactis*. RiPep2 is a 12-residue lanthipeptide with a small ring at its C-terminus (Figure 4A), which is formed after sequential modification of the precursor peptide by two enzymes (NisB and NisC). The dehydration by NisB decreases the mass of the peptide by 18 Da and can be detected in MS measurements. However, the detection of the cyclization reaction by NisC is cumbersome, as the reaction does not shift the mass or charge of the peptide (Figure S9). Therefore, conventional MS measurements cannot be used to detect the presence of the lanthionine ring and reactions with sulfhydryl reactive agents followed by MS are necessary to assess, indirectly, ring formation. First, we measured RiPeP2-Dhb (MW = 1464 Da, a linear peptide) and RiPep2 (MW = 1464 Da, a cyclized peptide) in octameric FraC^G13F^ nanopores. The peptides were measured separately (Figure S10) and in a mixture (Figure 4C). RiPep2 induces deeper blockades (84.2 ± 1.4 *I*_ex_%) compared to the linear peptide RiPep2-Dhb (79.6 ± 1.5 *I*_ex_%), a difference that can also be detected in a mixture of the peptides with an *R_s_* of 3.17 (Table S2). Hence, differences in constitutional isomers can also be detected by nanopore recordings. The nanopore measurements of RiPep2 also yielded events that almost fully blocked the nanopore (*I*_ex_% > 95%), which are not observed in the measurement of RiPep2-Dhb. These likely originate from small contaminants in the sample resulting from the purification of the peptide from *L. lactis* or from the subsequent modification by NisB and NisC.

We then tested SyncA2, a 17-residue peptide with an 11 amino acid ring (Figure 4B), a remarkable aspect in lanthipeptide research.^35^ The dehydration and cyclization of the peptide are both catalyzed by SyncM, a newly characterized enzyme. The dehydrase activity of SyncM can be monitored directly by MS measurements; however, the cyclase activity cannot, as it does not change the mass of the peptide. The SyncA2 peptides are larger than the RiPep2 peptides and are expected to fully block octameric FraC^G13F^ nanopores. Therefore, we used octameric FraC^Wt^ nanopores. We measured SyncA2 (MW = 1732 Da, Figure S11) and a synthetic peptide (SyncA2-L, MW = 1748 Da) that mimics the SyncA2 peptide before dehydration and cyclization. The peptides were measured separately (Figure S12) and in a mixture (Figure 4D). The linear synthetic peptide induced short ionic current blockades in the ionic current trace, but the cyclized peptide SyncA2 yielded a remarkably different type of blockade, with longer dwell times (Figure S13) and with large ionic current fluctuations during the event (Figure 4D). Despite the clear difference in event features, we could not fully separate the events in the *I*_ex_% vs dwell time spectrum (Figure S12). It has been reported before that 3D mapping of the events, using also the ionic current fluctuations during the nanopore events, can improve the separation of peptide clusters.^36^ Therefore, we classified the events by their *I*_ex_%, dwell time, and σ_b_, the standard deviation of the ionic current during the event. Using logistic regression, we classified the events based on these features, with a true positive rate (TPR) of 90.2% for SyncA2-L and 75.9% for SyncA2 (Figure S14). The low TPR for SyncA2 might be a result of the presence of some linear peptide in the sample, as a peak corresponding to the mass of SyncA2-L was also present in the MS spectrum (Figure S11). In addition, some dehydrated peptides could be present, which would have the same mass as SyncA2. Therefore, we also measured SyncA2-C (MW = 1730 Da, a linear peptide that mimics SyncA2 before the cyclization reaction, Figure S12) in order to exclude the differences in ionic current fluctuations being a result of the dehydration reaction. We observed a nanopore signal that is similar to SyncA2-L, confirming that the difference in nanopore signal between SyncA2 and SyncA2-L was due to the cyclization reaction rather than the dehydration step. Even though the majority of SyncA2 events could be classified, more advanced event detection methods have been reported that might further capture the event characteristics of the high-variance events of SyncA2 peptides.^37^ In combination with machine learning approaches,^38,39^ this may allow for an even better discrimination between cyclic and unmodified peptides.

In order to study whether we can quantify the extent of cyclization, we compared the detection frequencies of RiPeP2 and RiPep2-Dhb, as these peptides were best separated in the nanopore spectrum. Interestingly, we found that the cyclized peptide RiPeP2 has a significantly higher detection rate than the unmodified peptide in an equal molar mixture (Table S3), indicating that the cyclized peptide is either more efficiently captured or more easily detected by the nanopore. The bulky ring structure likely increases the probability of the peptide interacting with the nanopore, which may increase the probability of detection. Although such differences in capture or detection frequency complicate the precise quantification of the lanthionine ring formation, quantification is still possible as described in earlier work.^23^ Using this method, we estimated that about 71% of the peptides were cyclized (Table S3).

## Conclusions

Nanopore spectrometry is emerging as a technique to measure small molecules at the single-molecule level and its applicability toward the detection of homopolymers^9,40^ and peptides^12,41^ has been demonstrated. The technique is simple and robust, and nanopores can be integrated into low-cost portable devices. In addition, the nanopore signal is tuned by interactions between the analyte and the nanopore. This is an important feature of nanopore analysis, as it suggests that in principle all physicochemical features in molecules can be addressed.

In this work, we have demonstrated the ability of biological nanopores to discriminate between constitutional isomers, enantiomers, and diastereomers that are difficult or impossible to be detected directly by conventional mass spectrometry alone. First, we showed that the nanopore signals of L11 and D11, two 11-residue enantiomeric peptides, were distinguishable in the CytK and FraC nanopores. Interestingly, CytK^Wt^ could not discriminate between the two peptides, but two mutants where residue Lys128 was substituted could.

Molecular dynamics simulation revealed that the difference in the nanopore signal most likely originated from different chiral interactions of the peptides during translocation across the nanopore. The engineering of the inner lumen of nanopores with aromatic residues improved molecular recognition by increasing the residence time of the peptides and by inducing a more compact structure within the nanopore. Interestingly, in FraC nanopores the *I*_ex_% of D11 was higher than L11, opposite to what was observed in CytK nanopores. This could indicate a difference in chiral interaction of these peptides with the FraC nanopore compared to that of CytK nanopores. However, the methods used were customized with beta-barrel pores in mind, limiting the direct transferability of the method to other systems. Moreover, we show that our nanopore system is sensitive enough to detect a difference of only one *D*-amino acid in enkephalin peptides obtaining an approximately 82% separation.

Finally, we measured two sets of lanthionine peptides and showed that lanthionine ring formation shifts the nanopore signal. Ring formation does not change the *m*/*z* of the peptides and cannot be detected by conventional MS directly. Typically, these assays are performed with sulfonyl reactive agents followed by MS and/or LC-MS/MS, which requires lengthy additional experiments and/or expensive facilities. Here we show that ring formation can be assessed within a few minutes. In our experiments, we observed a preferred capture of cyclic peptide over unmodified peptide. By correcting for this difference in capture frequency, we could directly quantify the extent of ring formation from the nanopore spectrum. Measurements of other lanthipeptides might help us to understand and predict the differences in capture and detection frequency of the peptides in the future. Hence, nanopore technology presents exciting possibilities for the rapid and cost-effective analysis of peptides, even those that cannot be differentiated by mass spectrometry.

## Materials and Methods

### Expression of His6-Tagged Peptides in *L. lactis* NZ900

First, an inoculum of *Lactococcus lactis* NZ900 strains containing two plasmids (Table 1), one with the modification enzyme (*nis*BC or *syncM*) and one with the substrate (*RiPep2* or syncA2), both under the nisin promoter, was made. The initial culture was set in 50 mL of liquid medium M17 + 0.5% glucose (GM17) supplemented with 5 μg/mL chloramphenicol and 10 μg/mL erythromycin and grown overnight at 30 °C, without shaking, and was afterward used as an inoculum for the coexpression of the enzymes with its specific substrate.

**Table 1:** Plasmids Used for the Expression of Lanthipeptides

pTLR-*syncM* pNZ8048-*syncA2*	Ery^R^, *Cm*^R^, s*yncA2 peptide* and *syncM* modification enzyme. Cloned individually under P_*nis*_ promoter in pNZ8048 in *L. lactis* NZ9000 pTLR-SyncM, respectively
RiPep2	ref (42)

For RiPep2, 1 L of minimal expression medium was inoculated with a 1:20 dilution of the overnight culture. For SyncA2 peptide, 4 L of GM17 (5% glucose, 5 μg/mL chloramphenicol, and 10 μg/mL erythromycin) was inoculated with a 1:50 dilution of the overnight culture. After that, the cultures were grown at 30 °C until an OD_600_ of 0.4 was reached. Peptide expression was induced with 5 ng/mL nisin; cultures were grown overnight under the same conditions. The purification of RiPep2 was performed as previously described.^42^

### Purification of Cyclized SyncA2

Culture with *Lactococcus lactis* (pTLR-SyncM pNZ8048-SyncA2) was harvested by centrifugation (4 °C, 8000 rpm, 40 min), the supernatant was discarded, and the pellet was resuspended in binding buffer (20 mM NaH_2_PO_4_, 0.5 M NaCl, 30 mM imidazole, pH 7.4). Then, the cells were disrupted by sonication (VibraCell, 30 s ON, 10 s OFF, 75% amplitude). Lysates were centrifuged, and the peptides were purified from the supernatant using a column filled with 4 mL of Ni-NTA agarose (Qiagen). Peptide was eluted with 5 mL of elution buffer (20 mM NaH_2_PO_4_, 500 mM NaCl, 500 mM imidazole, pH 7.4). The 5 mL elution from the affinity chromatography step was divided into 1 mL fractions and further purified using a reverse-phase C18 (Phenomenex Aeris 250 × 4.6 mm^2^, 3.6 μm particle size, 100 Å pore size) Agilent Infinity HPLC system. The sample was filtered through a 0.2 μm filter (Phenomenex) before being loaded on the HPLC column. The column was equilibrated in 5% solvent B (100% acetonitrile (ACN):0.1% trifluoroacetic acid (TFA)) and in solvent A (ultrapure water with 0.1% TFA), with a first step of 5% (solvent B) for 10 min, and then a linear gradient of 20–100% (solvent B) was applied to elute the peptides at a flow rate of 1 mL/min. Fractions containing peaks were collected and analyzed by matrix-assisted laser desorption/ionization with a time-of-flight detector (MALDI-TOF). Next, fractions with full peptide (leader + SyncA2 core) were freeze-dried and resuspended in 1 mL of 50 mM ammonium acetate (Sigma-Aldrich) adjusted to pH 6.5. Later, 100 μL of NisP protease was added to the resuspended samples and incubated at 37 °C for 2 h to release the SyncA2 core peptide. Finally, we performed a second HPLC run with 10 min of 5% (solvent B) followed by a linear gradient (from 25 to 40%, solvent B) HPLC to obtain the pure core peptide. We collected and analyzed the peak fractions by MALDI-TOF in reflector mode. The core peptide eluted between 34 and 38% ACN. HPLC samples with SyncA2 core peptide were freeze-dried and resuspended in 100 μL of Milli-Q water.

A small amount of sample was analyzed by MS. First, HPLC samples (1 μL) were spotted on a target and dried at room temperature. Then, 1 μL of a α-cyano-4-hydroxycinnamic acid matrix (3 mg/mL) was spotted over the samples, and the matrix was dried at room temperature. The analysis was made using a 4800 plus MALDI/TOF analyzer (Applied Biosystems) operated in MS reflector mode.

### FraC Nanopore Purification

Plasmid containing the gene for His_6_-tagged FraC was transformed by electroporation into *E. coli* BL21 cells and grown overnight on an LB-agar plate supplemented with 1% glucose and 100 μg/mL ampicillin. On the next day, the colonies were pooled and grown in 200 mL of 2YT medium at 37 °C until an OD_600_ of 0.6–0.8 was reached. At that point, the production of FraC was induced by the addition of 0.5 mM IPTG. After induction, the culture was incubated overnight at 25 °C, and the cells were afterward harvested by centrifugation (4000 rpm, 20 min) and stored at −80 °C for at least 30 min. Cell pellets from 200 mL of cell culture were resuspended in 20 mL of lysis buffer (150 mM NaCl, 15 mM Tris, 20 mM imidazole, 1 mM MgCl_2_, 10 μL DNaseI, and approximately 1 mg of lysozyme) for 40 min at room temperature under constant rotation. Afterward, the cells were fully disrupted by sonication using a Branson Sonifier 450 (2 min, duty cycle 30%, output control 3) and cell debris was pelleted by centrifugation (6000 rpm, 20 min). Meanwhile, approximately 200 μL of Ni-NTA bead solution (Ni-NTA agarose, Qiagen) was washed with wash buffer (150 mM NaCl, 20 mM imidazole, 15 mM Tris, pH 7.5). The supernatant was transferred to a fresh falcon tube and mixed with the bead solution for 5 min at room temperature under constant rotation. The supernatant solution was then loaded on a Micro Bio-Spin column (Bio-Rad) and subsequently washed with 5 mL of wash buffer. The protein was eluted in fractions of 250 μL using elution buffer (150 mM NaCl, 300 mM imidazole, 15 mM Tris, buffered to pH 7.5). FraC monomers were oligomerized using 1:1 (m:m) DPhPC:sphingomyelin liposomes. The protein fractions were pooled and incubated in a 1:10 protein:liposome ratio at 37 °C for 1 h. The liposomes were afterward disrupted by the addition of 0.6% LDAO. The disrupted liposomes were diluted in 10 mL of buffer containing 150 mM NaCl, 20 mM imidazole, 15 mM Tris (pH 7.5), and 0.02% DDM. The solution was then incubated with 200 μL of prewashed Ni-NTA bead solution (Ni-NTA agarose, Qiagen) for 5 min at room temperature under constant rotation, loaded on a Micro Bio-Spin column (Bio-Rad), and subsequently washed with 5 mL of wash buffer (150 mM NaCl, 20 mM imidazole, 15 mM Tris (pH 7.5), and 0.02% DDM). Oligomers were eluted in fractions of 250 μL using an elution buffer containing 150 mM NaCl, 1 M imidazole, and 15 mM Tris, buffered to pH 7.5. FraC oligomers can be stored in the refrigerator for several weeks.

### Purification of CytK Nanopores

Plasmid containing the gene for His_6_-tagged CytK was transformed by electroporation into *E. coli* BL21 cells and grown overnight on an LB-agar plate supplemented with 1% glucose and 100 μg/mL ampicillin. On the next day, the colonies were pooled and grown in 200 mL of 2YT medium at 37 °C until an OD_600_ of 0.6–0.8 was reached. At that point, the production of CytK was induced by the addition of 0.5 mM IPTG. After induction, the culture was incubated overnight at 25 °C and the cells were then harvested by centrifugation (4000 rpm, 20 min) and stored at −80 °C for at least 30 min. Cell pellets from 200 mL of cell culture were resuspended in 20 mL of lysis buffer (150 mM NaCl, 15 mM Tris, 20 mM imidazole, 1 mM MgCl_2_, 0.02% DDM, 10 μL DNaseI, and approximately 1 mg of lysozyme) for 40 min at room temperature under constant rotation. Afterward, the cells were fully disrupted by sonication using a Branson Sonifier 450 (2 min, duty cycle 30%, output control 3) and cell debris was pelleted by centrifugation (6000 rpm, 20 min). Meanwhile, approximately 200 μL of a Ni-NTA bead solution (Ni-NTA agarose, Qiagen) was washed with wash buffer (150 mM NaCl, 20 mM imidazole, 0.02% DDM, and 15 mM Tris, pH 7.5). The supernatant was transferred to a fresh falcon tube and mixed with the bead solution for 5 min at room temperature under constant rotation. The supernatant solution was then loaded onto a Micro Bio-Spin column (Bio-Rad) and subsequently washed with 5 mL of wash buffer. The protein was eluted in fractions of 250 μL using elution buffer (150 mM NaCl, 300 mM imidazole, 0.02% DDM, and 15 mM Tris, buffered to pH 7.5). After purification, CytK nanopores can directly be used in electrophysiology experiments or stored in the refrigerator for several months.

### Planar Lipid Bilayer Recordings

Nanopore recordings were performed in a chamber consisting of two compartments separated by a 25-μm-thick Teflon (Goodfellow Cambridge Ltd.) membrane. The Teflon membrane contained an aperture with a diameter of approximately 100 μm. First, 5 μL of 5% hexadecane in pentane was applied near the aperture. The pentane was left to dry, and afterward 400 μL of buffer (1 M KCl, 50 mM citric acid, titrated with bis-tris propane to pH 3.8 or 3 M LiCl, 50 mM citric acid, buffered to pH 3.8) was added to both compartments. Then, approximately 10 μL of a 10 mg/mL solution of DPhPC dissolved in pentane was added on top of the buffer on each side of the chamber. The chamber was left to dry shortly to allow the evaporation of pentane, and finally Ag/AgCl electrodes were inserted into each compartment. The *cis* compartment was connected to the ground, and the *trans* compartment to the working electrode. Planar lipid bilayers were formed by repeatedly lowering and raising the buffer in each compartment until a stable bilayer with a capacitance of approximately 100 pF was formed.

### Nanopore Measurements of Isobaric Peptides

A tiny amount of FraC or CytK oligomers (typically 1–10 ng) was added to the *cis* compartment, and the bilayer was broken and reformed until a single channel was present in the bilayer. The orientation of the nanopore could be detected by the asymmetry in the current–voltage relation. A 2 min blank recording was measured, and afterward the substrate (e.g., D11, L11, or a mixture of both peptides) was added to the *cis* or *trans* compartment. The measurement of the peptides was then recorded for at least 2 min.

### Data Acquisition

The ionic current traces were recorded using a Digidata 1440A (Molecular Devices) connected to an Axopatch 200B amplifier (Molecular Devices). All measurements were recorded with a sampling frequency of 50 kHz and with a Bessel filter of 10 kHz.

### Event Detection

Data analysis was performed using Clampfit software (Molecular Devices). The open nanopore current (*I*_0_) and the noise in the open nanopore current (σ_*I*_0__) were determined from the ionic current trace. First, the histogram of the ionic current was taken, and a Gaussian was fitted around the open nanopore current. *I*_0_ is determined from the center of the peak, and σ_*I*_0__, from the standard deviation of the peak. Then events were detected using a threshold search with a threshold of 5*σ_*I*_0__, and only events with a minimum duration of 50 μs were collected. The excluded current percent (*I*_ex_%) was calculated using , where Δ*I*_B_ is the magnitude of the current blockade as calculated by the Clampfit software.

### Classification of Peptides from Nanopore Spectra

The event clusters belonging to D11 and L11 were identified by the sequential addition of peptides to the nanopore. The event clusters of the enkephalin and RiPep peptides were identified by the sequential addition of peptides to the nanopore. To calculate the overlap between event clusters, the mean and spread of *I*_ex_% were calculated from individual peptide measurements. The resolution (*R_s_*) was then calculated by using eq 1.

### Event Classification of the SyncA2 Peptide Measurements Using Logistic Regression

Events were classified using the Classification Learner from Statistics and Machine Learning Toolbox 12.3 in MATLAB. First, a data set with event characteristics (*I*_ex_%, dwell time, and σ_b_) of events observed in the single peptide measurements of SyncA2 and SyncA2-L was constructed. From this data set, we extracted events that satisfy 50% < *I*_ex_% < 90%. This selected data set was then used to train the Classification Learner using a logistic regression model. The trained model was then used to predict the events from the measurements of a mixture of SyncA2 and SyncA2-L.

### Quantification of the Extent of RiPep2 Modification

The extent of cyclization of RiPep2 was quantified by measuring the relative event frequency of RiPep2 events in the RiPep2 sample and in a mixture of RiPep2 and RiPep2-Dhb. First, we counted the events belonging to RiPep2 and RiPep2-Dhb in the spectra by considering all events that satisfy μ(*I*_ex_%) – σ(*I*_ex_%) < *I*_ex_% < μ(*I*_ex_%) + σ(*I*_ex_%), where the values of μ(*I*_ex_%) and σ(*I*_ex_%) can be found in Table S2. We then calculated the relative detection factor using
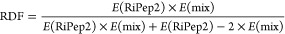
2where *E*(RiPep2) is the percentage of RiPep2 events in the modified sample and *E*(mix) is the percentage of RiPep2 events in the mixture. The extent of modification (conversion) was then estimated using
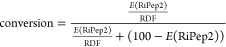
3

### Molecular Dynamics Simulations

#### System Setup

Exhaustive simulations of peptide conformational dynamics alone are technically challenging and computationally expensive; therefore, a balance had to be found for sampling as much of the relevant phase space of a translocation event and accuracy.^43^ To reduce the system size, the CytK nanopore was truncated to include only the barrel-forming part (residues 114 to 154). The shape of the barrel was kept in place by gentle positional restraints to the reference structure. The force of these restraints was tuned to match the flexibility of the same region in a full-size CytK embedded in a DOPC bilayer, measured over a 500 ns reference simulation (Figure S5). In lieu of an experimentally determined structure, the structure of the CytK nanopore is a homology model based on α-hemolysin.^32^ The K128F mutation was introduced using PyMOL^44^ and a subsequent relaxation of the side chains.

Starting configurations of the L11 peptide were also constructed with PyMOL. The d-amino acid version of the peptide, D11, was constructed in the same way, except that the *x* component of all coordinates was inverted and the residues were renamed. We then used simulated annealing^45^ to generate plausible initial conformations for the peptides.

An example of a representative initial condition of the simulation setup is shown in Figure S4. All systems (either WT or a K128F truncated CytK nanopore with a L11 or D11 peptide) were put in a rectangular simulation box of average dimensions 4.7 × 3.9 × 10.1 nm^3^ and subsequently solvated in water (1970 molecules). K^+^ and Cl^–^ ions were added to a final concentration of 1 M, neutralizing the total charge of the system. Any titratable side chains were protonated as appropriate for pH 7.5. Each system underwent 1000 steps of energy minimization, followed by equilibration in NVT and NPT ensembles for 200 and 400 ps, respectively. Production runs were performed with metadynamics (see below).

#### Simulation Details

All MD simulations were carried out using GROMACS 2020.4 with a 2 fs integration time step, full periodic boundary conditions, and the CHARMM36m force field with the CHARMM-modified TIP3P water model.^46,47^ The pressure was coupled semi-isotropically to the Berendsen barostat at 1 bar, in which the *z* axis was made incompressible to avoid changing the length of the box in that axis, and without reference to coordinate scaling to maintain the shape of the barrel.^48^ Temperature control was carried out at 303.15 K using the v-rescale algorithm.^49^ van der Waals forces were calculated with a cutoff of 12 Å and a switching distance of 10 Å. Electrostatics were calculated using a plain cutoff with a radius of 12 Å so as not to introduce unnatural long-range effects from neglecting the membrane in the simplified simulations.

### Metadynamics

To enhance the sampling of the peptide conformations and locations inside the nanopore, we used well-tempered metadynamics.^33^ To this end, GROMACS was patched with the open-source, community-developed PLUMED library, version 2.7.^50,51^ As a collective variable to drive the enhanced sampling, we used the *z* position of the center-of-mass of the peptide in the pore. The *z* position was calculated in scaled units, ranging from −0.5 to 0.5, from the bottom to the top of the simulation box. The initial height of the Gaussians was 1.2, with a σ of 0.02 and a bias factor of 20. The Gaussians were deposited on a grid with automatic bins between −0.5 and 0.5.

A number of additional biases were applied to direct the conformational space sampled by the peptides. To prevent misalignment of the peptide when entering the pore, the peptide structure was gently biased toward staying linear and aligned with the pore. This was done using the lower and upper wall functionality in PLUMED such that once the radius of gyration would dip below a threshold of 0.8 nm, a bias would be applied to stay above the threshold. The same was done to limit the offset in the *xy* plane (i.e., moving away from the central pore axis) calculated between the Cα atoms of the first and last residues of the peptide. The threshold for this was set at 0.5 nm, and a bias was applied to keep this offset below the threshold. The peptide was always initially aligned with the pore, with the N-terminus pointing down to mimic the alignment to the electric field that would be present in electrophysiology experiments.

We simulated the peptide translocation events of both D11 and L11 in CytK^Wt^ and CytK^K128F^ nanopores. For each system, we performed six replicas of 100 ns, thereby exhaustively sampling the peptide location and configurations inside the pore. Using this setup, a single pass of the peptide through the pore is completed in under 100 ns.

### Analysis

To construct the free-energy landscape of peptide translocation, the metadynamics positional bias was then reweighed together with the bias of the radius of gyration and the orientation. The energy landscape was calculated in terms of the *z*-axis position of the center of mass of the peptide and the radius of gyration of its backbone atoms. For the density plots, the positions of the Cα-atoms of the peptide were recorded over time on a 3D lattice and reweighed to unbias the trajectory. The lattice was then smoothed and visualized as a volumetric map using PyMOL. The visualization of the initial configuration was done by taking the final frame of the 500 ns reference simulation and making all but the truncated β-barrel and the peptide transparent.

## Supporting Information Available

Measurements of individual peptides, voltage dependency measurements, raw ionic current traces, starting conformation of MD simulations, mass spectrometry measurements of lanthipeptides, and calculation of the resolution of the nanopore (PDF)
