# Supplementary material for: Seeing the Invisibles: Detection of Peptide Enantiomers, Diastereomers, and Isobaric Ring Formation in Lanthipeptides Using Nanopores
Source: J Am Chem Soc. 2023 Aug 14;145(33):18355–65. doi: 10.1021/jacs.3c04076 (PMC10450680; doi:10.1021/jacs.3c04076)
Supplement: Supplementary file 1 — ja3c04076_si_001.pdf [file ja3c04076_si_001.pdf]

Seeing the invisibles: detection of peptide enantiomers, diastereomers and isobaric ring formation in lanthipeptides using nanopores

Roderick Corstiaan Abraham Versloot, Patricia Arias-Orozco, Matthijs Jonathan Tadema, Florian Leonardus Rudolfus Lucas, Xinghong Zhao, Siewert J. Marrink, Oscar Paul Kuipers, Giovanni Maglia[*]

Groningen Biomolecular Sciences and Biotechnology Institute, University of Groningen, 9747AG, Groningen, Netherlands

* Correspondence: g.maglia@rug.nl

Table of contents

Figure S1: Additional measurements of D11 and L11 in CytK nanopores Page 3

Figure S2: Sequential addition of peptides to the nanopores Page 4

Figure S3: Voltage dependency measurements of L11 and D11 in CytK pores Page 5

Figure S4: Raw ionic current traces of D11 and L11 in FraC and CytK nanopores Page 6

Figure S5: Starting conformation and position restraints during MD simulations Page 7

Figure S6: Smoothened energy landscapes of the simplified simulations Page 8

Figure S7: Sequential addition of enkephalin peptides in FraC and CytK nanopores. Page 9

Figure S8: I_{ex}% vs σ_b plots for the enkephalin peptides Page 10

Figure S9: Mass-spectrometry measurement of RiPep2 and Ripep2-Dhb Page 11

Figure S10: Measurements of individual RiPep peptides Page 12

Figure S11: Mass-spectrometry measurement of SyncA2 peptide after purification. Page 13

Figure S12: Measurements of individual SyncA2 peptides and a mimic of dehydrated SyncA2 Page 14

Figure S13: Dwell time histograms of individual SynCA2 peptides Page 15

Figure S14: Logistic regression of SyncA2 peptide events Page 16

Table S1: Peptide Resolution of the nanopores for the Enkephalin peptides Page 17

Table S2: Resolution of the nanopore for the RiPeP peptides Page 17

Table S3: Quantification of the RiPeP peptide events Page 17

List of chemicals used in the methods Page 18

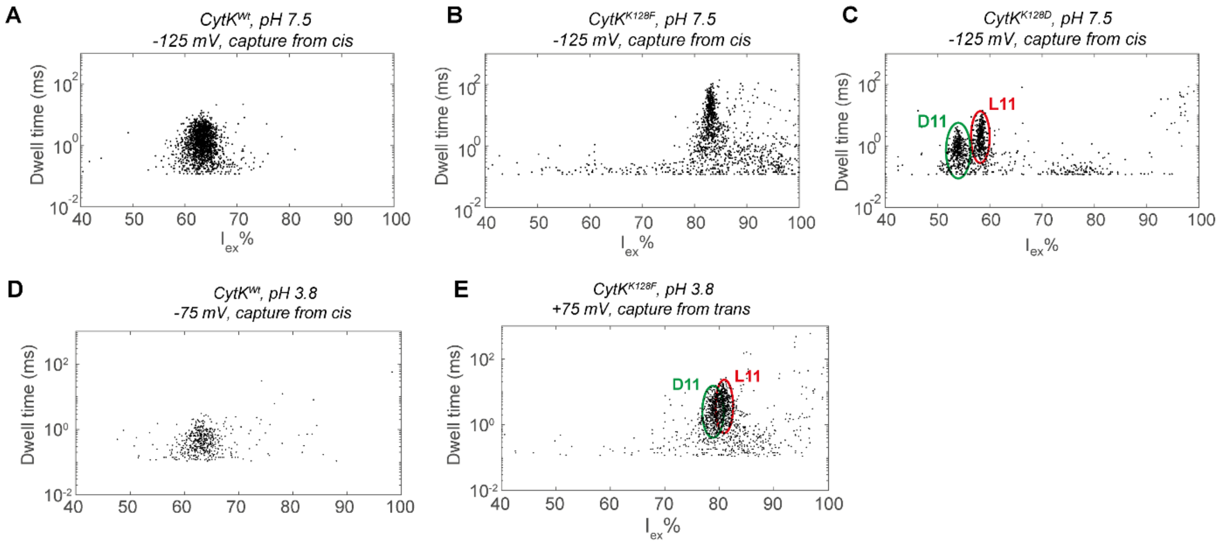

Figure S1: Measurements of D11 and L11 in CytK nanopores under higher applied voltage or at low pH. (A-C) measurement of a 1:1 (v:v) mixture of L11 and D11 at -125 mV applied potential in (A) CytK^{Wt}, (B) CytK^{K128D} and (C) CytK^{K128F}. The buffer consisted of 1 M KCl and 50 mM Tris buffered to pH 7.5 (D) measurement of 1:1 (v:v) mixture of L11 and D11 in CytK^{Wt} in 1 M KCl and 50 mM citric acid buffered to pH 3.8 using bistrispropane. (E) measurement of 1:1 (v:v) mixture of L11 and D11 added to the *trans* compartment of a CytK^{K128F} nanoproes in 1 M KCl and 50 mM citric acid buffered to pH 3.8 using bistrispropane.

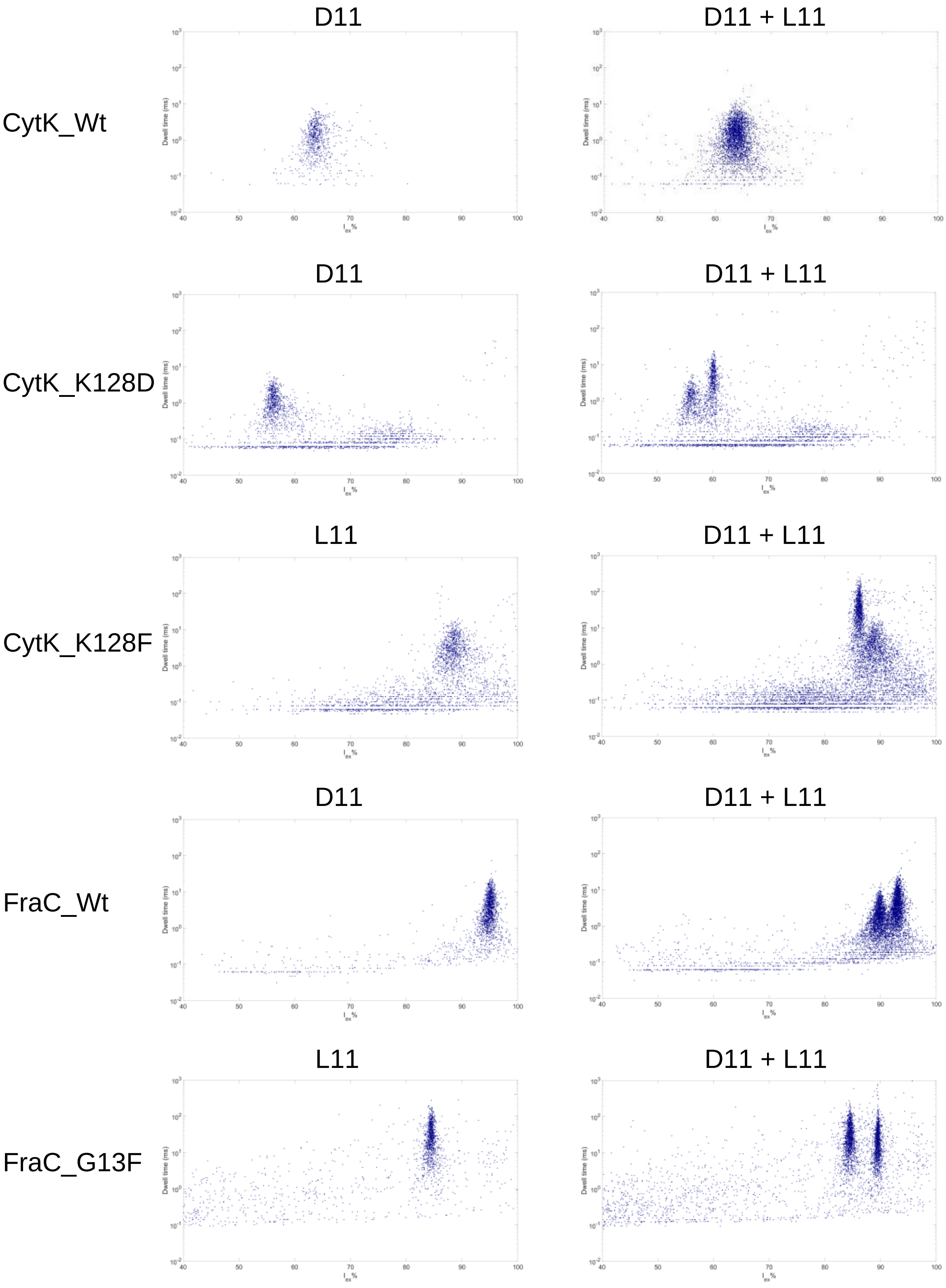

Figure S2: Sequential addition of peptides to the nanopores. The left panels show the nanopore spectrum of a single D- or L-peptide and the right panels show the nanopore spectrum after the addition of the second peptide to the same nanopore. CytK nanopores were measured in 1 M KCl buffered to pH 7.5 using Tris with -100 mV applied voltage, FraC nanopores were measured in 1M KCl with 50 mM citric acid, buffered to pH 3.8 using bistrispropane with -50 mV applied voltage.

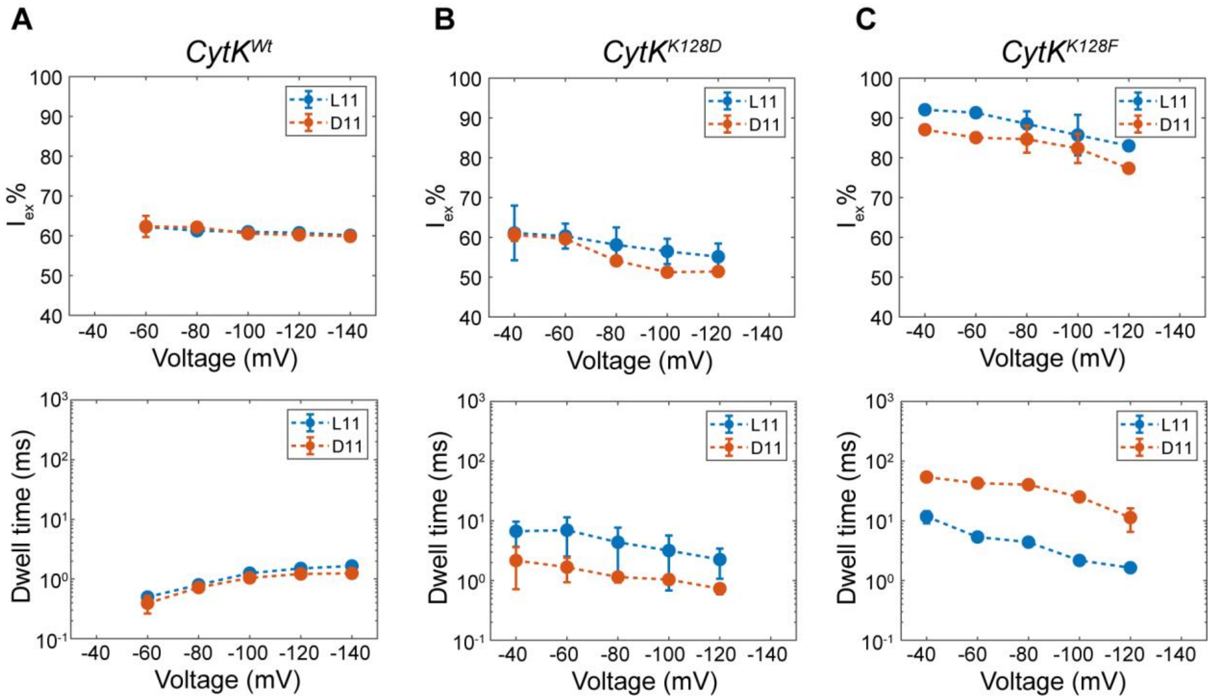

Figure S3: Voltage dependency of the I_{ex}% and dwell time of L11 and D11 in $CytK^{Wt}$, $CytK^{K128F}$ and $CytK^{K128D}$. Peptides L11 and D11 were measured separately at different voltages. 5 µM of either L11 or D11 was added to the *cis* chamber and the average I_{ex}% (top) and dwell time (bottom) of the translocation events was determined for (A) $CytK^{Wt}$, (B) $CytK^{K128D}$, and (C) $CytK^{K128F}$. Measurement in 1 M KCl and 50 mM Tris buffered to pH 7.5 with a sampling rate of 50 kHz and a 10 kHz Bessel filter.

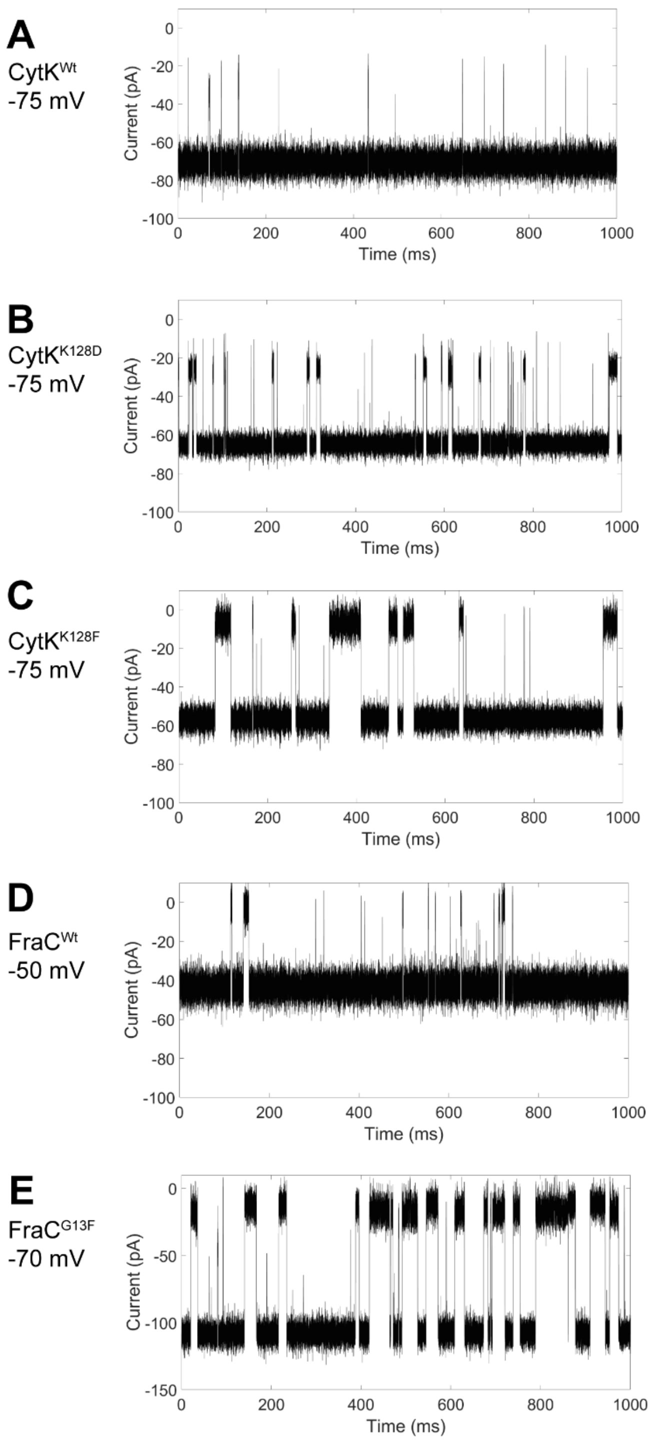

Figure S4: Raw ionic current traces of a mixture of D11 and L11 in FraC and CytK nanopores. (A-E**)** One second ionic current traces (50 kHz sampling frequency, 10 kHz Bessel filter) of a 1:1 (v:v) mixture of D11 and L11 peptides. Typical events and the event spectra are shown in Figure 1. CytK nanopores were measured in 1 M KCl buffered to pH 7.5 using Tris, FraC nanopores were measured in 1M KCl with 50 mM citric acid, buffered to pH 3.8 using bistrispropane.

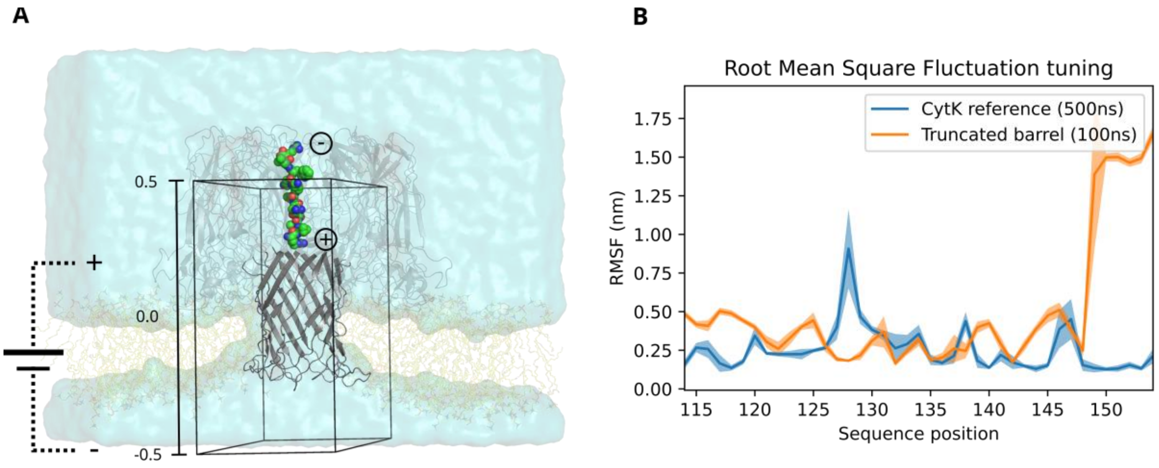

Figure S5: Starting conformation and position restraints during MD simulations
(A) A representative starting configuration for the molecular dynamics simulations. All the elements that were removed from the simplified simulation are made transparent. The pore barrel is centered in the box. The peptide is aligned along the axis of the pore and translated to be fully in solution. Note that the peptide crosses the periodic boundary and is thus simultaneously at the top and at the bottom of the barrel. The z-axis coordinates are indicated as scaled units so as to be consistent with all analysis. The experimentally applied potential is shown across the bilayer, but no electric field was present in the simulations (B) The position restraints on the truncated barrel were tuned to capture the magnitude of fluctuations in the reference barrel. The outline represents the standard deviation. The RMSF of the truncated barrel was measured in the presence of 10 kJ/mol position restraints. Because a single position restraint force was used throughout, the flexibility of the hydrophilic loops at the end of the barrel is overly dampened (position 128), while the truncated beta-strands that normally connect to the rest of the protein (beyond position 148) are too flexible. Given that the peptides sample mostly the interior part of the barrel, further optimization of these external regions was not attempted.

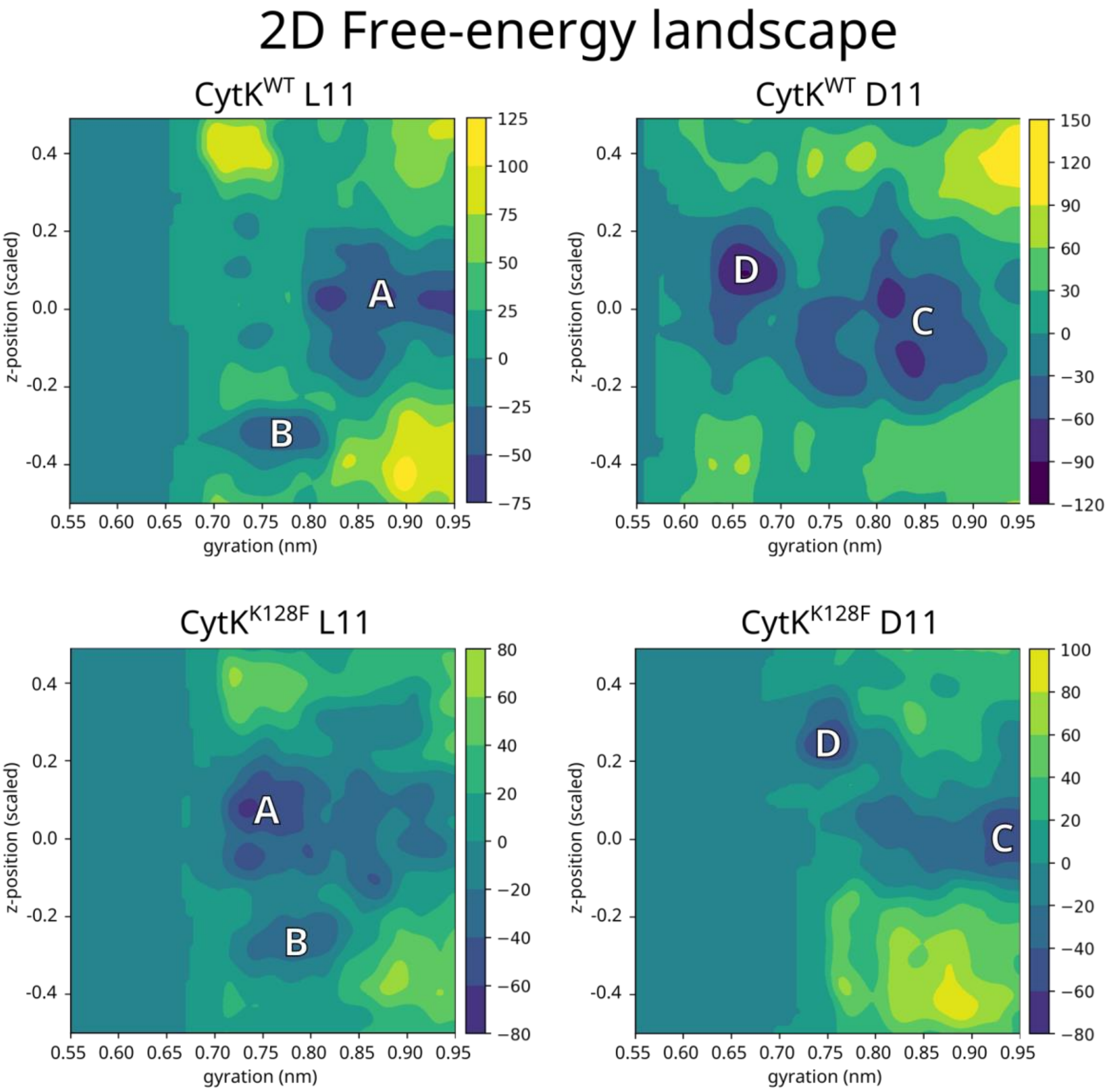

Figure S6: Smoothened energy landscapes of the simplified simulations. The z-position represents the center of mass of the peptide's backbone atoms from the bottom to the top of the simulation box (from -0.5 to 0.5 respectively). The truncated pore is centered in the box around 0.0 with the bottom of the pore around -0.3 and the truncated top around 0.3. The colors represent free energy in kJ/mol. A and B denote energy minima specific to the L11 peptide, while C and D represent energy minima specific to the D11 peptide. The mutant causes a shift to a lower radius of gyration in minimum A, but not B, whereas both C and D appear to shift towards a higher radius of gyration.

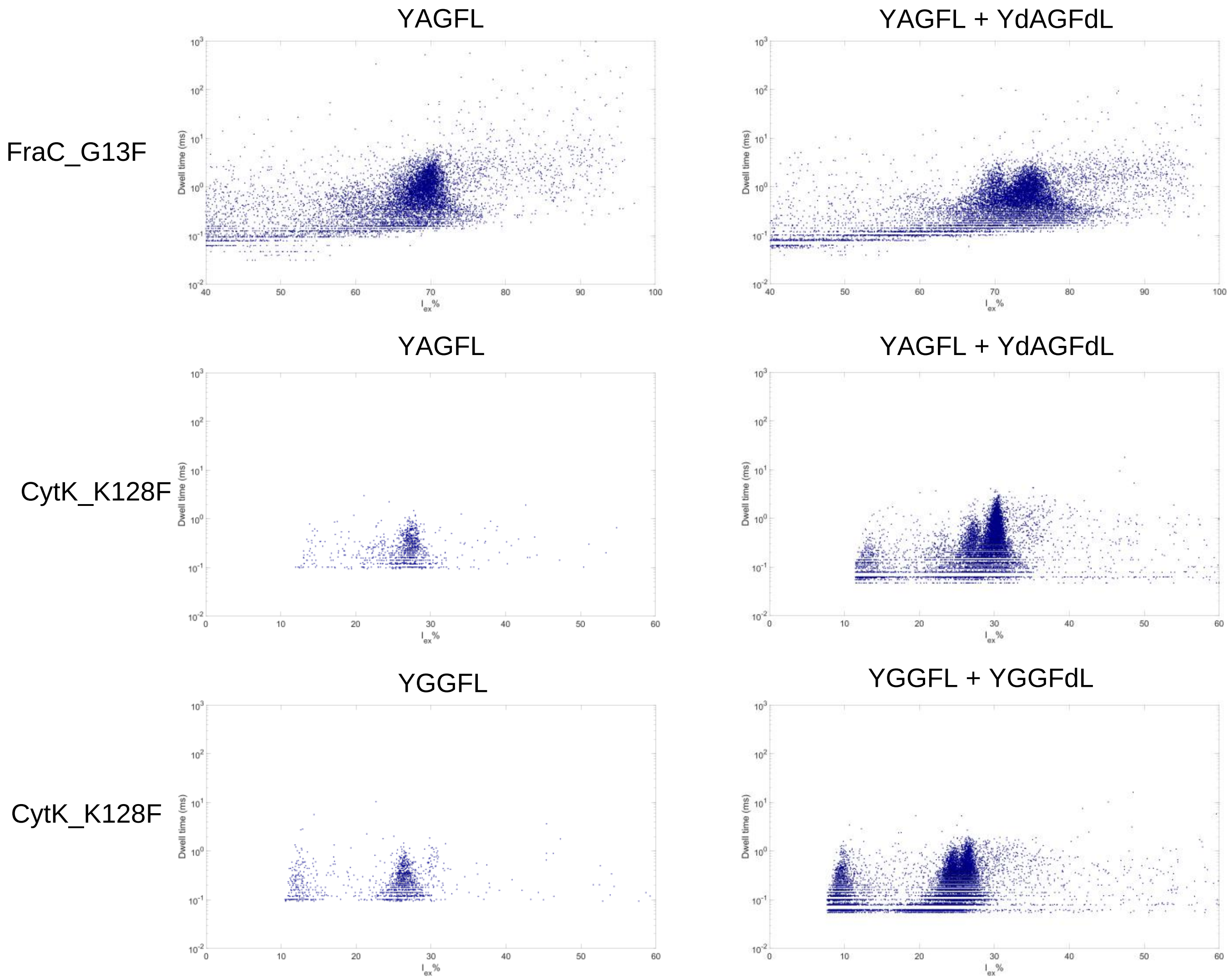

Figure S7: Sequential addition of enkephalin peptides in FraC and CytK nanopores. The left panels show the nanopore spectrum of a single Enkephalin and the right panels show the nanopore spectrum after the addition of the second Enkephalin peptide to the same nanopore. FraC nanopores were measured in 3M LiCl at pH 3.8 with an applied voltage of -70 mV. CytK nanopores were measured in 3M LiCl at pH 3.8, +100 mV applied voltage. Measurements with a sampling rate of 50 kHz and a 10 kHz Bessel filter.

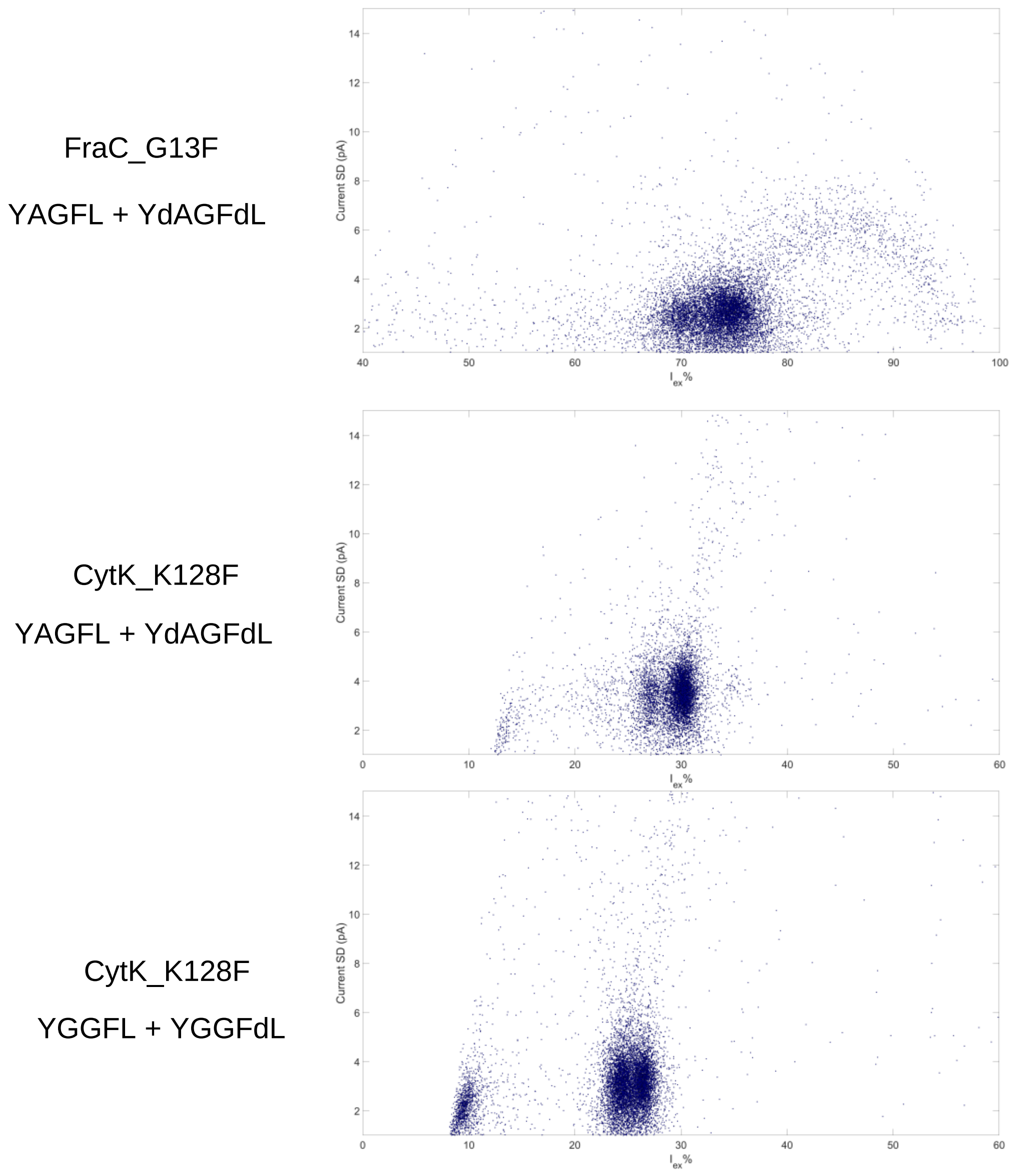

Figure S8: I_{ex}% vs σ_b (Current SD) plots for the enkephalin peptides. FraC nanopores were measured in 3M LiCl at pH 3.8 with an applied voltage of -70 mV. CytK nanopores were measured in 3M LiCl at pH 3.8, +100 mV applied voltage. Measurements with a sampling rate of 50 kHz and a 10 kHz Bessel filter.

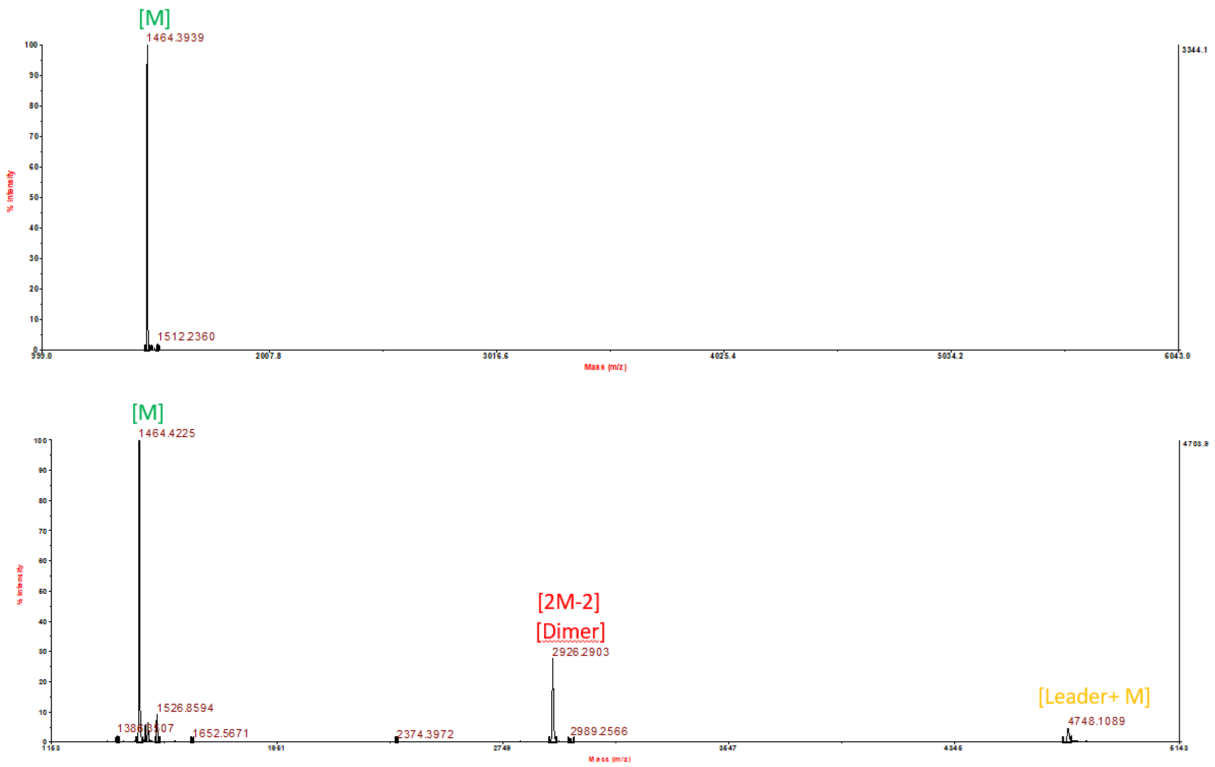

Figure S9: Mass-spectrometry trace of RiPep2 and Ripep2-Dhb. MALDI-TOF measurements of RiPep2 (top) and Ripep-Dhb (bottom). Both peptides have a mass of approximately 1464 Da. In the dehydrated peptide, some dimers were observed, most likely due to the formation of disulfide bridges. In addition, a small amount of leader peptide was detected.

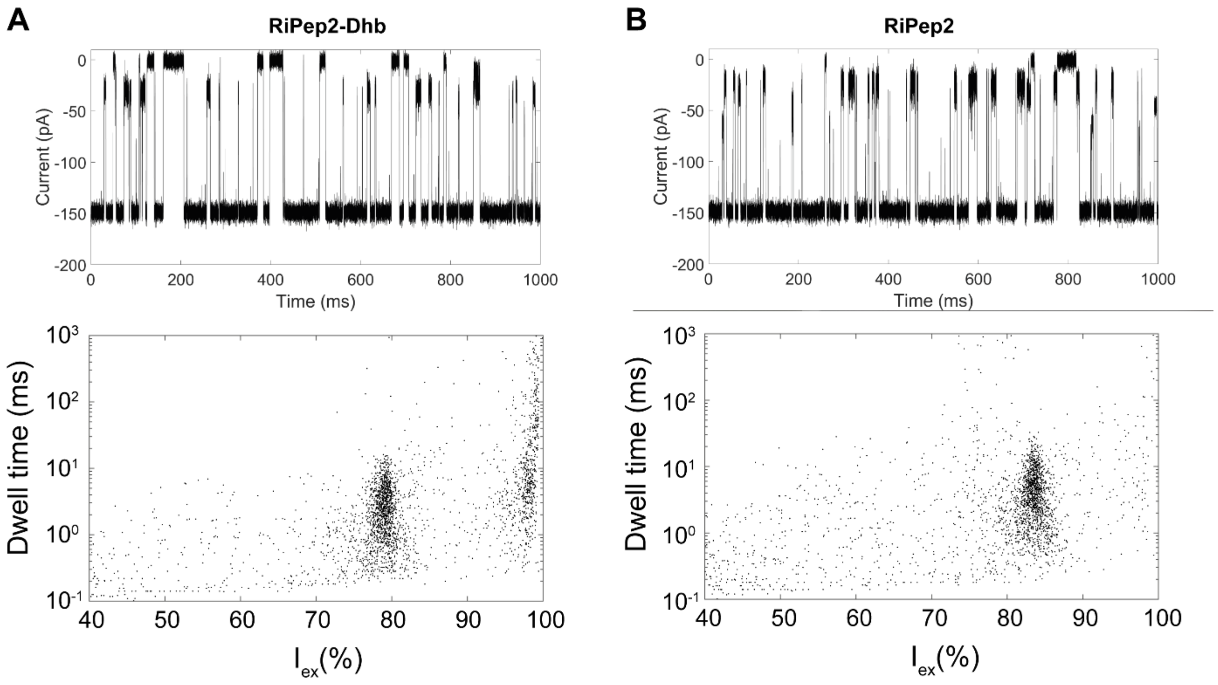

Figure S10: Measurements of individual RiPep peptides. Ionic current trace (top) and event spectrum (bottom) after the addition of (A) RiPep2-Dhb or (B) RiPep2 to an octameric FraC^{G13F} nanopore. Measurements in 1M KCl, pH 3.8.

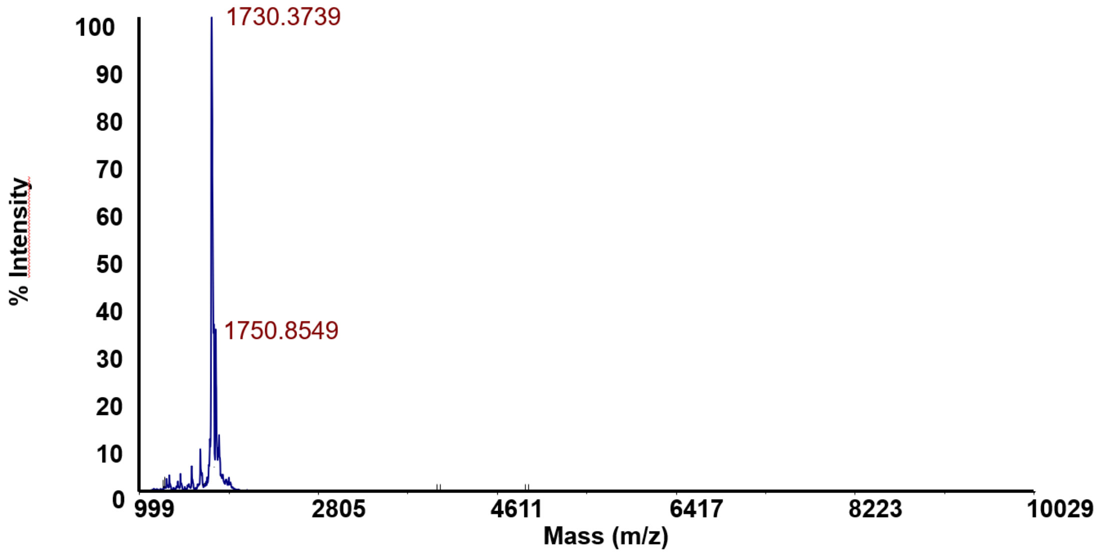

Figure S11: Mass-spectrometry trace of SyncA2 peptide after purification. MALDI-TOF measurements of SyncA2, with an expected mass of 1730 Da. An additional peak corresponding to unmodified peptide (approx. 1750 Da) was also observed.

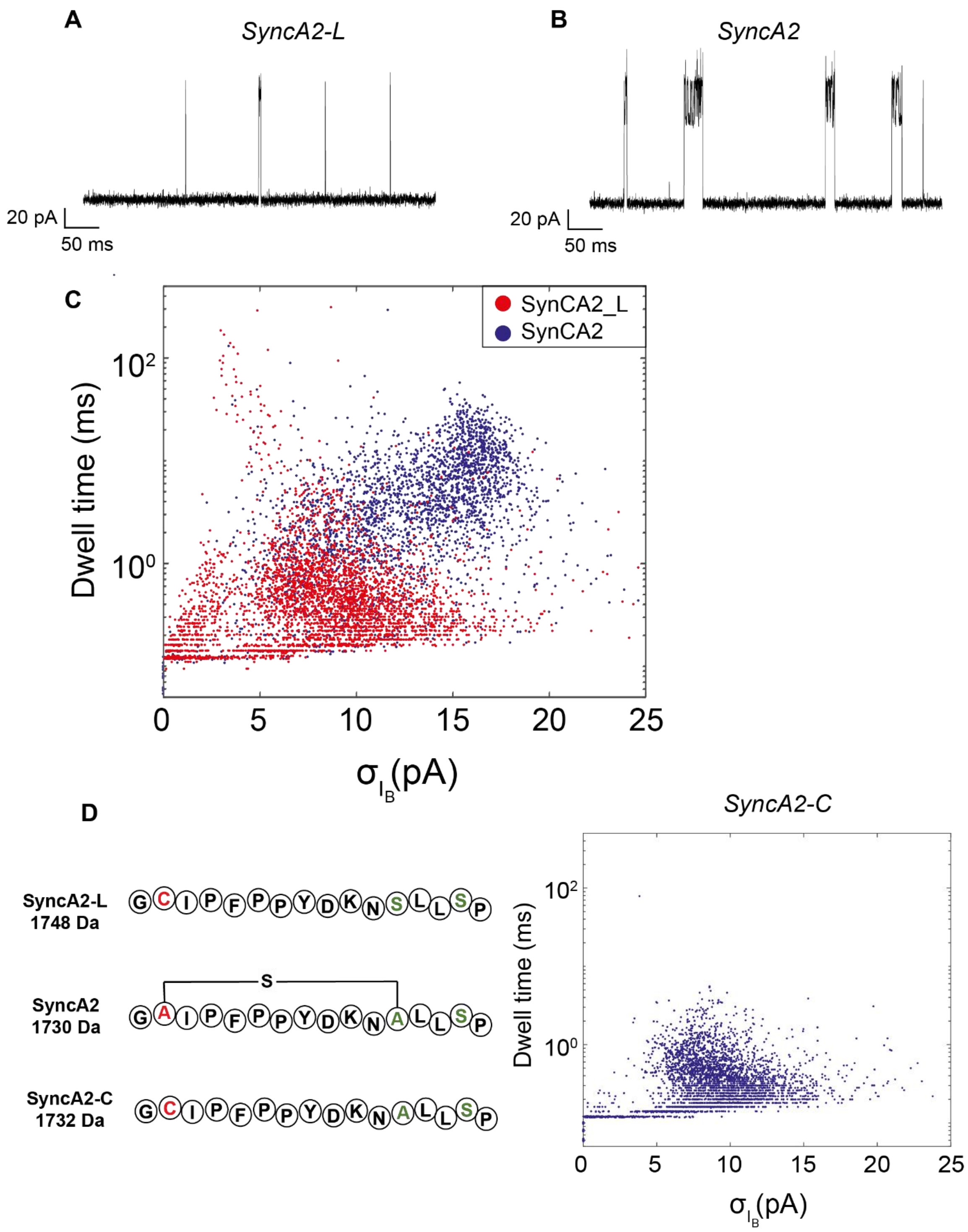

Figure S12: Measurements of individual SyncA2 peptides and a mimic of dehydrated SyncA2. (A) Ionic current trace showing short-lived blockades after the addition of SyncA2-L to FraC^{Wt}. (B) Ionic current trace showing long blockades with high ionic current fluctuations after the addition of SyncA2-L to FraC^{Wt}.(C) Overlaid spectrum of the SynCA2_L and SynCA2 spectrum (D) Schematic structures of SyncA2-L, SyncA2 and the control peptide SyncA2-C that mimics the dehydrated SyncA2. Nanopore spectrum (right) showing events that are similar to the SyncA2-L peptide shown in (A). Measurements in 1M KCl at pH 3.8.

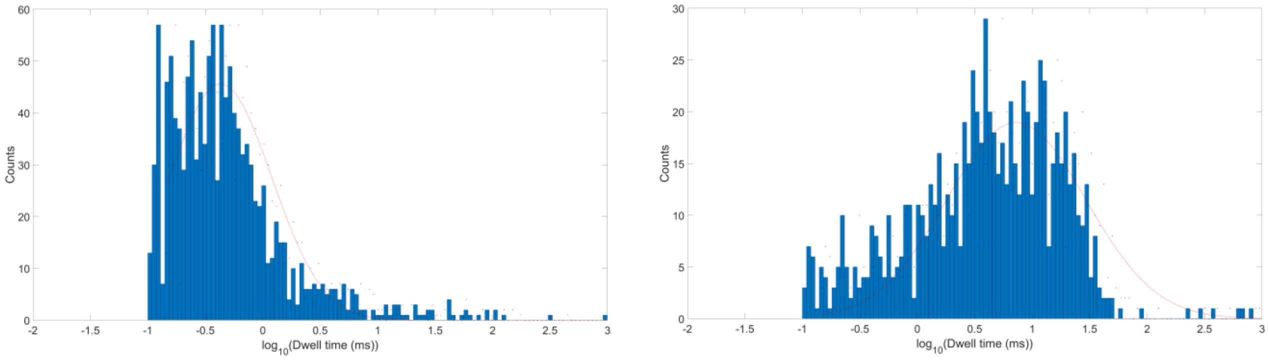

Figure S13: Dwell time histograms of individual SyncA2 peptides. Dwell time histograms of SyncA2-L (left) and SyncA2 (right) of measurements of individual peptides shown in Figure S12. The red line shows a gaussian fitting through the histograms.

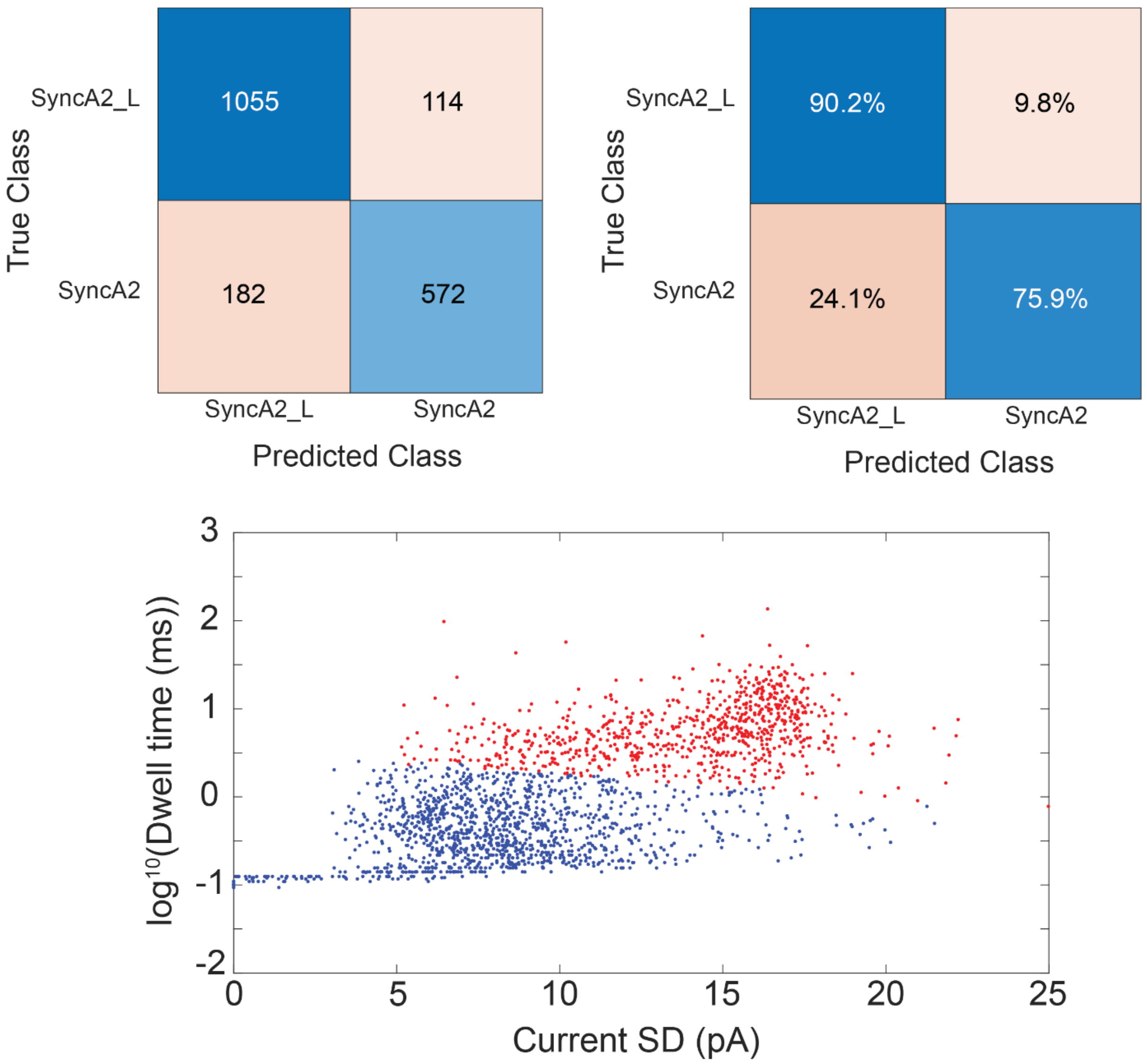

Figure S14: Logistic regression of SyncA2 peptide events. Classification matrix of total event counts (top left) and relative counts (top right) after logistic regression of the training data set. Scatter plot (bottom) showing the σ_b vs log^{10}(dwell time) plot of the measurement of a mixture of SyncA2 and SyncA2-L. Events classified as SyncA2 and SyncA2-L are indicated in red and blue, respectively.

Nanopore	Peptide	μ(I_{ex}%)	σ(I_{ex}%)	Rs
FraC_G13F	YAGFL	70.8	2.6	1.47
	YdAGFdL	74.4	2.3	
CytK_K128F	YAGFL	27.1	1.1	3.10
	YdAGFdL	30.2	0.9	
CytK_K128F	YGGFL	24.5	1.1	1.80
	YGGFdL	26.3	0.9	

Table S1: Peptide Resolution of the nanopores for the Enkephalin peptide mixtures shown in Figure 3. μ(I_{ex}%) and σ(I_{ex}%) are derived from gaussian fitting to the I_{ex}% histogram of individual peptide measurements. R_s is calculated using formula (1).

Peptide	μ(I_{ex}%)	σ(I_{ex}%)	Rs
RiPep2-Dhb	79.9	1.5	3.17
RiPep2	84.5	1.4	

Table S2: Resolution of the nanopore for the RiPeP peptides. μ(I_{ex}%) and σ(I_{ex}%) are derived from gaussian fitting to the I_{ex}% histogram of individual peptide measurements. R_s is calculated using formula (1).

Sample	Events RiPep2-Dhb	Events RiPep2	E(RiPep2)	RDF	Conversion
RiPep2	241	1604	86.9%	2.65	71.4%
1:1 mixture	902	1331	59.6%		

Table S3: Quantification of the RiPeP peptide events. The number of RiPep2-Dhb and RiPep2 events within μ(I_{ex}%) ± σ(I_{ex}%) were counted based on the values shown in Table S2. The RDF was calculated using formula (2) and the conversion was calculated using formula (3).

List of chemicals used in the methods

Potassium chloride (>99.5%), sodium chloride (>99.5%), lithium chloride (>99%), magnesium chloride (>98.5%), imidazole (>99%), dodecyl-β-D-maltoside (DDM, >99%), TRIS hydrochloride PUFFERAN® (≥99%), urea, LB medium and 2YT medium were purchased from Carl Roth. Bistrispropane (BTP, >99%), N,N-dimethyldodecylamine N-oxide (LDAO, >99%), pentane (>99%), n-hexadecane (>99%), lysozyme (from chicken egg white), chloramphenicol, erythromycin, nisin and glucose, ammonium acetate, alpha-Cyano-4-hydroxycinnamic, NaH2PO4, trifluoroacetic acid, and acetonitrile were purchased from Sigma-Aldrich. Citric acid (anhydrous, 99.6%) was obtained from Acros Organics. 1,2-Diphytanol-sn-glycero-3-phophocholine (DPhPC) lipids and sphingomyelin were obtained from Avanti. Ampicillin and isopropyl-β-D-1-thiogalactopyranoside (IPTG) were purchased form Fisher Scientific. M17 broth was purchased from Difco. Linear control peptides (SyncA2-L and SyncA2-C) were synthesized by Pepscan.
